# Environment-Sensitive Fluorescent Labelling of Peptides by Luciferin Analogues

**DOI:** 10.3390/ijms222413312

**Published:** 2021-12-10

**Authors:** Marialuisa Siepi, Rosario Oliva, Antonio Masino, Rosa Gaglione, Angela Arciello, Rosita Russo, Antimo Di Maro, Anna Zanfardino, Mario Varcamonti, Luigi Petraccone, Pompea Del Vecchio, Marcello Merola, Elio Pizzo, Eugenio Notomista, Valeria Cafaro

**Affiliations:** 1 Department of Biology, University of Naples Federico II, 80126 Naples, Italy; marialuisa.siepi@unina.it (M.S.); antonio.masino@unina.it (A.M.); anna.zanfardino@unina.it (A.Z.); varcamon@unina.it (M.V.); m.merola@unina.it (M.M.); elipizzo@unina.it (E.P.); vcafaro@unina.it (V.C.); 2 Department of Chemical Sciences, University of Naples Federico II, 80126 Naples, Italy; rosario.oliva2@unina.it (R.O.); rosa.gaglione@unina.it (R.G.); anarciel@unina.it (A.A.); luigi.petraccone@unina.it (L.P.); pompea.delvecchio@unina.it (P.D.V.); 3 Department of Environmental, Biological and Pharmaceutical Sciences and Technologies, University of Campania “Luigi Vanvitelli”, 81100 Caserta, Italy; rosita.russo@unicampania.it (R.R.); antimo.dimaro@unicampania.it (A.D.M.)

**Keywords:** fluorescent peptide, environment-sensitive fluorophore, peptide labeling, luciferin, membrane-binding peptide, antimicrobial peptide, antitumor peptide, RGD peptide

## Abstract

Environment-sensitive fluorophores are very valuable tools in the study of molecular and cellular processes. When used to label proteins and peptides, they allow for the monitoring of even small variations in the local microenvironment, thus acting as reporters of conformational variations and binding events. Luciferin and aminoluciferin, well known substrates of firefly luciferase, are environment-sensitive fluorophores with unusual and still-unexploited properties. Both fluorophores show strong solvatochromism. Moreover, luciferin fluorescence is influenced by pH and water abundance. These features allow to detect local variations of pH, solvent polarity and local water concentration, even when they occur simultaneously, by analyzing excitation and emission spectra. Here, we describe the characterization of (amino)luciferin-labeled derivatives of four bioactive peptides: the antimicrobial peptides GKY20 and ApoB_L_, the antitumor peptide p53pAnt and the integrin-binding peptide RGD. The two probes allowed for the study of the interaction of the peptides with model membranes, SDS micelles, lipopolysaccharide micelles and *Escherichia coli* cells. K_d_ values and binding stoichiometries for lipopolysaccharide were also determined. Aminoluciferin also proved to be very well-suited to confocal laser scanning microscopy. Overall, the characterization of the labeled peptides demonstrates that luciferin and aminoluciferin are previously neglected environment-sensitive labels with widespread potential applications in the study of proteins and peptides.

## 1. Introduction

Environment-sensitive fluorophores are fluorophores, the excitation spectra, emission spectra and/or quantum yields (QY) of which depend on variables such as pH, solvent polarity, viscosity and even molecular crowding/aggregation state. Several different molecular mechanisms can contribute to modulation of the fluorescence of environment-sensitive fluorophores [1]. Solvatochromic fluorophores, the λ_max_ of which changes with solvent polarity, are often push-pull molecules with an electron-donating and an electron-withdrawing group bound to an aromatic moiety. Solvent polarity strongly affects the intramolecular charge transfer of these molecules, thus modulating their fluorescence [1]. In a less common type of solvatochromic fluorophores, an excited-state intramolecular proton-transfer event generates tautomeric forms with different absorption and emission spectra [1]. In other cases, conformational changes or variations in the aggregation state strongly influence the fluorescence of the probe [1]. Finally, pH probes have two or more ionizations states with different fluorescence properties [2]. Often, only one of the pH-dependent species is fluorescent.

These fluorophores are very valuable tools in the study of a wide variety of molecular and cellular processes [1,2]. As protein and peptide labels, they find applications in the study of conformational variations of proteins, of protein/protein, protein/ligand and protein/membrane interactions but also in the design of biosensors [1,3,4]. For these reasons, the search for new environment-sensitive fluorescent probes and, in particular, for protein and peptide labels, is a very active research field.

**Figure 1:**
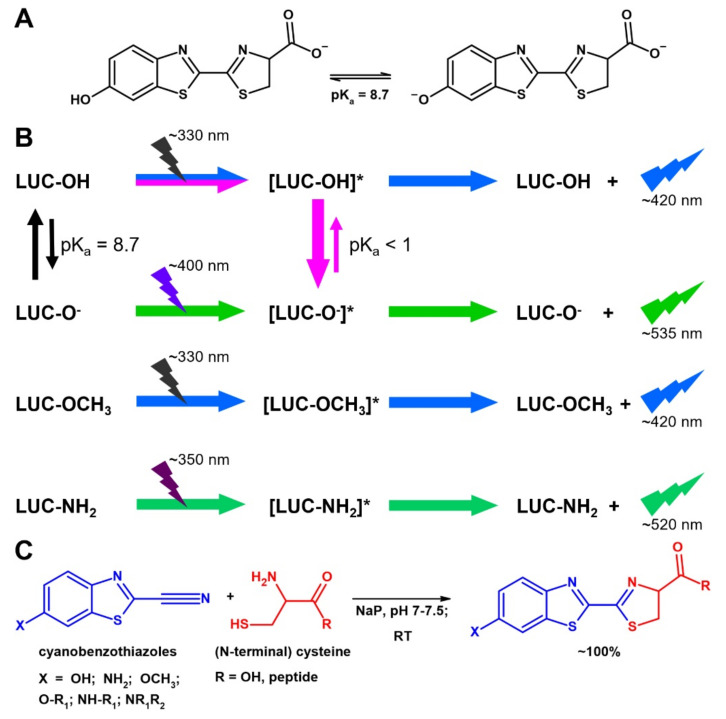
Structure, fluorescence and synthesis of luciferin and its analogues. (**A**) Structure of luciferin in phenol and phenolate forms. (**B**) Fluorescence of luciferin (Luc-OH), luciferin phenolate (Luc-O^−^), methoxyluciferin (Luc-OCH3) and aminoluciferin (Luc-NH2). Excited states are indicated by an asterisk. (**C**) Condensation reaction between 6-substituted 2-cyanobenzothiazoles and cysteine (free or as N-terminal residue of a peptide). R_1_ and R_2_ can be a wide variety of alkyl and acyl groups. The nature of R_1_ and R_2_ has little or no impact on the reaction but can strongly influence fluorescence.

Firefly luciferin (Luc), the substrate of firefly luciferase, is the most popular bioluminescent compound [5]. It is less known that Luc is also an environment-sensitive fluorophore with unusual properties, summarized in Figure 1. Even if Luc is typical push-pull molecule, the hydroxyl group at position 6 of the benzothiazole moiety behaves as a weak acid with a pK_a_ ≈ 8.7 (Figure 1A) [6]. Both the phenol and the phenolate forms are strongly fluorescent but with very different excitation and emission wavelengths (Figure 1B) [7]. However, as Luc is a “photoacid” [7,8,9], it undergoes a light-induced dissociation with a pK_a_ < 1 (Figure 1B). Thus, in the presence of water or other proton acceptors, Luc fluorescence is exclusively the result of the magenta and green pathways shown in Figure 1B. Even in neutral and acidic aqueous media, only the emission of the phenolate at 530 nm is generally observed, regardless of the excitation wavelength. Only in anhydrous organic solvents, e.g., acetonitrile or DMSO, does Luc show the blue fluorescence emitted by the phenol form (blue pathway in Figure 1B) [8,9]. However, very low amounts of water cause the appearance of the green emission. For example, in acetonitrile containing 14% water, the intensity of the blue and green emissions is comparable [8].

Two synthetic Luc analogues (Figure 1B), methoxyluciferin (mLuc) and aminoluciferin (aLuc), bearing non-dissociable groups at position 6 of the benzothiazole moiety show a single emission peak, with emission maxima similar to those of Luc phenol and phenolate forms, respectively [10,11]. mLuc is a very weak fluorophore [10], whereas aLuc is brighter than Luc itself; very interestingly, it is a strongly solvatochromic fluorophore with shifts up to 40 nm in the λ_max_ values [11].

In spite of these intriguing features, there are very few reports of probes exploiting (amino)luciferin fluorescence to detect cell thiols [12,13,14] or Furin activity [15]. To our knowledge, no example of direct fluorescent labeling of proteins or peptides based on Luc exists. This is even more surprising, considering that a Luc moiety can be incorporated very easily at the N-terminus of proteins and peptides. The final step of biological synthesis of Luc is a spontaneous condensation between D-cysteine and 6-hydroxy-2-cyanobenzothiazole (Figure 1C). This reaction is included among the so-called “click reactions” that are quantitative, irreversible and very fast in physiological conditions (phosphate buffer, pH 7–7.4, 25–37 °C) [16,17].

L-cysteine at the N-terminus of a peptide reacts with 2-cyanobenzothiazole (CBT) similarly to free cysteine, as the carboxyl group is not involved in the reaction (Figure 1C). Moreover, the nature of the substituent at position 6 of CBT also has a limited impact on the reaction. Therefore, peptides with an N-terminal cysteine residue are efficiently labeled trough a Luc-like spacer by using 6-substituted 2-cyanobenzothiazoles carrying any desired molecule (e.g., biotin, metal chelates, fluorophores, etc.) conjugated at position 6 (Figure 1C) [16,17]. In these bioconjugates, the label is usually attached through acylation of the amino group of aLuc or, less commonly, through alkylation of the hydroxyl group of Luc (Figure 1C), thus strongly reducing the fluorescence of (amino)luciferin [10,14,18]. Very interestingly, even if CBT reacts quantitatively and irreversibly with N-terminal cysteines, it generally forms a reversible adduct with the thiol group of internal cysteine residues, which can be removed by adding free cysteine or other aminothiols [16,17]. Therefore, an N-terminal cysteine can also be selectively labeled in the presence of cysteine residues at other positions on the peptide.

In order to study the possible exploitation of (amino)luciferin as an environment-sensitive fluorescent label for peptides, we synthesized (amino)luciferin conjugates of four peptides of different lengths, compositions and biological properties. Characterization of the fluorescence properties of the labeled peptides demonstrates that Luc and its analogues are previously neglected bright and photostable fluorescent labels suited for in vitro studies like peptide/membrane or peptide/cell interactions and microscopy/imaging studies.

## 2. Results

### 2.1. Preparation and Labeling of the Peptides

To evaluate the suitability of Luc and aLuc as environment-sensitive fluorescent labels, we selected four previously described bioactive peptides: GKY20 (20 residues), ApoB_L_ (37 residues), p53pAnt (37 residues) and RGD (6 residues) (Figure S1, Supplementary Materials). GKY20 [19] and ApoB_L_ [20] are cryptic cationic antimicrobial peptides (CAMP), peptides unusually rich in cationic and hydrophobic residues, which kill bacteria by damaging structural integrity and functions of bacterial membranes [21,22,23]. Moreover, CAMPs often show additional pharmacologically relevant biological activities, like antifungal, antitumor and anti-inflammatory activity [23,24]. p53pAnt (also spelled p53p-Ant) is a designed antitumor peptide containing a sequence at the N-terminus that induces apoptosis by interacting with p53, as well as a “cell-penetrating peptide” (CPP) at the C-terminus that derived from the Antennapedia protein homeodomain, which can drive peptides inside eukaryotic cells both by directly crossing the cytoplasmic membrane and by mediating endocytosis [25,26,27]. RGD is a natural ligand of integrins, cell surface proteins involved in adhesion to the extracellular matrix [28,29]. It is very different from the other three peptides; it is very short, hydrophilic and uncharged. RGD interaction with eukaryotic cell membranes depends on binding to a protein receptor rather than on a direct interaction with membrane lipids, as in the case of CAMPs and CPPs. Immobilized RGD-like peptides are frequently used to promote the adhesion of eukaryotic cells to surfaces, polymers, hydrogels, etc. Moreover, they may find applications in diagnostic and imaging fields [30,31]. Peptides GKY20, ApoB_L_ and p53pAnt with an additional cysteine residue at the N-terminus—herein named (C)GKY20, (C)ApoB_L_ and (C)p53pAnt—were prepared using a recently described strategy for the preparation of recombinant toxic peptides in *E. coli* based on the selective cleavage of a carrier/peptide fusion bearing the acid sensitive sequence Asp-Cys [32] (details in Supplementary Materials, Sections S2–S4). Peptide RGD with an additional cysteine residue at the N-terminus—(C)RGD—was obtained by chemical synthesis. The four peptides were labeled with either a Luc or an aLuc moiety at the N-terminus by incubation with a slight molar excess of 6-hydroxy-2-cyanobenzothiazole or 6-amino-2-cyanobenzothiazole, respectively, in aqueous buffer at pH 7.4 at 25 °C (Supplementary Materials, Section S5). As expected, based on the well-known high efficiency of this reaction, Luc-labeled peptides (Luc-GKY20, Luc-ApoB_L_, Luc-p53pAnt and Luc-RGD) and aLuc-labeled peptides (aLuc-GKY20, aLuc-ApoB_L_, aLuc-p53pAnt and aLuc-RGD) were obtained with yields close to 100% after very short incubation times (30–60 min). (C)GKY20 was also treated with 6-methoxy-2-cyanobenzothiazole to obtain an mLuc-labeled peptide (mLuc-GKY20) and with PyMPO maleimide, the thiol-reactive version of PyMPO, a widely used photostable solvatochromic fluorophore [33,34,35], to obtain the peptide PyMPO-(C)GKY20. After purification (Section 4 and Supplementary Materials), the mass of all labeled peptides was confirmed by MALDI-MS (Table S1). SDS-PAGE analysis of labeled GKY20, ApoB_L_ and p53pAnt shows their bright fluorescence under UV light (365 nm) and their solvatochromic nature appreciable even by the naked eye (Figure S2).

### 2.2. Stability and Biological Activity of Luc-Labeled Peptides

When choosing the best fluorophore to label a peptide, in addition to excitation and emission wavelengths, other relevant parameters are (photo)stability and molecular features, which might influence the biological activity of the peptide to be labeled (molecular weight, charge, hydrophobicity, etc.).

Although Luc is a fairly stable molecule, in order to evaluate the photostability of Luc-labeled peptides, we monitored the fluorescence of Luc-GKY20, aLuc-GKY20 and mLuc-GKY20 for 60 min, constantly exciting the three labeled peptides at their respective maximum absorption. As comparative control, we used the very photostable fluorophore PyMPO [33] by treating PyMPO-(C)GKY20 in the same conditions. Luc-GKY20 lost only 30% of its initial fluorescence (Figure S3), thus proving to be even more photostable than PyMPO-(C)GKY20, which lost 43% of its initial fluorescence. aLuc-GKY20 and mLuc-GKY20 proved to be slightly less photostable than Luc-GKY20, with a loss of initial fluorescence of about 55% and 60%, respectively. Therefore, Luc can be considered a very stable label, though aLuc is also a suitable tool.

An ideal fluorescent label or probe should not significantly change the properties (size, hydrophobicity, charge, etc.) of the peptide to be labeled in order to minimize the risk of altering its chemical-physical behavior and/or biological activity. Therefore, we compared molecular weight, accessible surface area (ASA), polar ASA, ratio of polar ASA/ASA and number of charged groups (Table S2) of Luc, aLuc and a wide panel of commonly used solvatochromic and non-solvatochromic fluorescent labels that can be attached either at the N-terminus of a peptide (generally through an amide or a sulfonamide bond) or at the side-chain sulfur of a cysteine residue (generally through a thioether bond). Only NBD and some coumarin dyes among fluorescent labels that can be attached at the N-terminus and only bimane among fluorescent labels that can be attached to the side chain of a cysteine residue have an ASA smaller than that of Luc and aLuc. Moreover, several solvatochromic and not-solvatochromic fluorophores are either quite hydrophobic (e.g., pyrene-1-butirrate, Atto 495, most BODIPY labels, BADAN etc.) or very hydrophilic and with several charged groups (e.g., Alexa Fluor™ 405 and Alexa Fluor 488-C5-maleimide), whereas Luc and aLuc have an intermediate ratio of polar ASA/ASA (0.31–0.34) and no (aLuc) or low charge (Luc at neutral pH). Therefore, Luc and aLuc can be confidently considered fluorescent labels with a low impact on the molecular properties of the labeled peptide.

In order to determine the influence of Luc labels on the biological activity of the peptides considered in this study, we compared the antimicrobial activity of labeled GKY20 and ApoB_L_ with that of the corresponding unlabeled recombinant peptides, (P)GKY20 [36] and (P)ApoB_L_ [20] (Supplementary Materials, Section S6). The cytotoxic activity of labeled and unlabeled p53pAnt was also investigated (Supplementary Materials, Section S7). The minimum inhibitory concentrations (MIC) of Luc-GKY20 and aLuc-GKY20 were found to be identical to those measured for (P)GKY20 on Gram-negative and Gram-positive strains (Table S3). The MIC values of Luc-ApoB_L_ and aLuc-ApoB_L_ were found to be very similar to those measured for (P)ApoB_L_ (the observed differences, not higher than one dilution, are generally considered not significant in the microdilution assay).

Luc-p53pAnt and (C)p53pAnt showed similar toxicity when assayed on two human cell lines, namely HaCaT noncancerous immortalized keratinocytes and HeLa cervical cancer cells (Figure S4). It is worth noting that labeled and unlabeled p53pAnt showed significantly higher toxicity for HeLa cancer cells than for HaCaT cell, as expected of an anticancer peptide.

Finally, we tested the effect Luc-RGD and unlabeled RGD on HaCaT and HeLa cells. As expected, we did not observe any significant toxic effect.

### 2.3. Response of the Labeled Peptides to Solvent Polarity Changes

Most solvatochromic fluorophores show an increase in fluorescence emission and a blue shift of the λ_max_ as solvent polarity decreases. Therefore, we measured emission spectra and QY of the labeled peptides in sodium phosphate 10 mM, pH 7.4 (NaP) and in the same buffer containing 50% (*v*:*v*) either isopropanol or methanol.

The emission spectra in NaP of the Luc- and aLuc-labeled peptides showed maximum emission at 539 and 526 nm, respectively (Figure S5 and Table 1). As expected, all the labeled peptides showed a blue shift of the λ_max_ in the presence of the organic solvents (Table 1). The blue shift was less in the case of Luc-labeled peptides (6–7 nm) and greater in the case of aLuc-labeled peptides (9–15 nm). Furthermore, aLuc-labeled peptides showed a considerably greater blue shift in isopropanol than in methanol, thus confirming that aLuc is a probe more sensitive to solvent polarity compared to Luc. mLuc-GKY20 showed blue-shift values very similar to those of aLuc-GKY20, whereas PyMPO-(C)GKY20 showed a 5 nm blue shift only in the presence of isopropanol.

Intriguingly, emission intensities (Figure S5) and QY values (Table 2) showed complex and partially unexpected variation characteristic of each peptide. For example, all the variants of GKY20 showed very large increases in emission intensities and QY values in the presence of the organic solvent (2.5–6 times higher in 50% isopropanol than in water). On the contrary, Luc-p53pAnt and aLuc-p53pAnt showed a 30–35% reduction in QY. Both labeled variants of ApoB_L_ and RGD showed little or no variation in emission intensities (Figure S5) and QY (Table 2). These puzzling variations are likely the result of a very well-known phenomenon, i.e., the quenching of fluorophores bound to protein/peptides. Even if several mechanisms can contribute to quenching, the most common is photoinduced electron transfer (PET), a reversible light-triggered transfer of electrons from amino-acid residues to the fluorophore [37]. The most efficient donors are tryptophan and tyrosine, although to a lesser extent, histidine and methionine can also contribute significantly to quenching [38]. Obviously, the redox potential of a fluorophore and therefore its propensity to accept electrons, influences its sensitivity to quenching [37].

PET is also very sensitive to distance and orientation of the donor/acceptor couple, so the quenching efficiency can be influenced even by minor variations in the conformation of the labeled protein/peptide. In fact, PET-mediated quenching is a powerful tool for detection of conformational variations in proteins and peptides [37].

Very interestingly, the peptides that show the largest variations, namely GKY20 and p53pAnt, contain several residues with strong quenching ability (tryptophan, histidine and two tyrosines in GKY20; two tryptophans, three histidines and a methionine in p53pAnt; Figure S1).

The QY values of the Luc-labeled peptides were also measured in sodium acetate at pH 5.0 to induce the protonation of the histidine residues. Protonation of histidine residue can influence PET quenching both directly, by reducing the donor ability of the imidazole ring, and indirectly, by inducing conformational changes. As expected, significant increases in QY values were observed only for the three peptides containing histidine residues: GKY20, p53pAnt and ApoB_L_ (Table 2). To date, it has not been possible to determine the relative contribution of the direct and indirect effect of histidine protonation on quenching.

### 2.4. Response of Luc-Labeled Peptides to pH

Luc has a phenolic hydroxyl, which behaves as a weak acid, with a pK_a_ of about 8.7 [6]. As Luc and its phenolate have quite different excitation spectra, with maxima at 330 and 395 nm, respectively, the excitation spectra of the Luc-labeled peptides recorded at pH 7.4 show a characteristic shoulder at 390–400 nm originating from the small amount of phenolate ion present at this pH (Figure S6). It should be noted that the shoulder is also visible in the presence of organic solvents (Figure S5). On the other hand, the shoulder disappears in buffers with pH values below 6, as Luc becomes protonated, whereas the peak at 400 nm prevails at pH values greater than 8.5 (Figure S6). By recording the emission at 539 nm of the Luc-labeled peptides after excitation at 400 nm in buffers with different pH values, we determined the actual pK_a_ values of the Luc moieties (Table 3). All pK_a_ values were found to be slightly lower than those of free Luc, with small differences among the peptides, likely due to differences in the net charge and distribution of positively charged residues. In particular, the highest pK_a_ value was found for Luc-RGD, the only peptide with a negative net charge at pH values close to the pK_a_ of the Luc moiety (Table 3). The remaining three peptides have a high positive net charge, which could stabilize the phenolate anion, thus lowering the pK_a_ value. The possibility of selectively exciting the phenolic or phenolate form of Luc not only makes Luc a useful pH probe for the pH range of 7–8 but also allows for monitoring of the formation of peptide/ligand complexes in which the ionization of the Luc moiety is suppressed or altered. The next sections show some applications of this peculiar feature of Luc.

### 2.5. Interaction of Labeled Peptides with Liposomes

Both CAMPs and CPPs are able to interact with biological membranes, and this interaction is essential to their properties. Therefore, in order to verify whether Luc-labeling can be used to investigate peptide/membrane interaction, we studied the behavior of the labeled peptides in the presence of liposomes composed either of pure palmitoyl-oleoyl-phosphatidylcholine (POPC) or of a mixture of POPC and palmitoyl-oleoyl-phosphatidylglycerol (POPG) at a molar ratio of 4:1. As the POPC head group is zwitterionic, liposomes composed only of this lipid are neutral and are usually considered mimetic of eukaryotic cell membranes. On the contrary, POPG-containing liposomes are negatively charged and are considered a simplified model of bacterial cell membranes.

**Figure 2:**
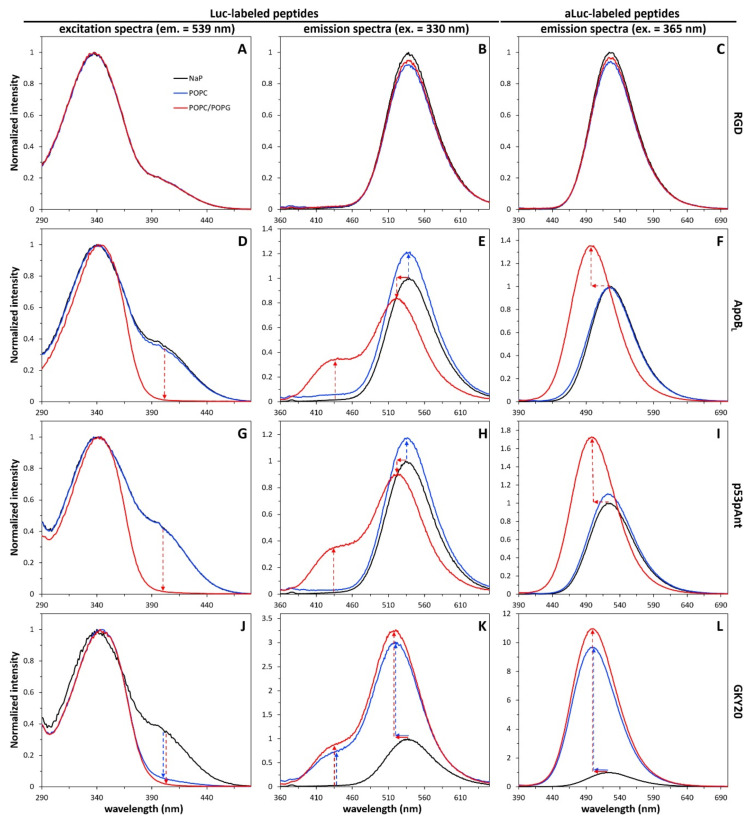
Fluorescence of labeled peptides in the presence of liposomes of POPC or POPC/POPG (5:1). Excitation spectra of luciferin (left), emission spectra of luciferin (center) and emission spectra of aminoluciferin (right) were recorded for peptides RGD (**A–C**), ApoB_L_ (**D–F**), p53pAnt (**G–I**) and GKY20 (**J–L**). Spectra recorded in the presence of liposomes were normalized to the corresponding spectra in NaP. Arrows highlight the main changes with respect to NaP.

The short, hydrophilic and negatively charged labeled RGD was used as a negative control. As expected, the excitation and emission spectra of Luc-RGD were essentially identical in NaP and in the presence of the two types of liposomes (Figure 2). 

The same result was obtained in the case of the emission spectrum of aLuc-RGD (Figure 2). On the contrary, in the case of Luc-ApoB_L_, the excitation spectrum in the presence of POPC/POPG liposomes was clearly different from the spectra in NaP and in the presence of POPC liposomes, completely lacking a shoulder at 400 nm (Figure 2). This suggests that in the presence of POPC/POPG liposomes, the deprotonation of the Luc phenolic group is inhibited. Two mechanisms could explain this finding: (i) embedding of the phenolic group of Luc among the lipids would directly prevent deprotonation; or (ii) binding of the Luc probe to the surface of the negatively charged POPC/POPG liposomes could prevent deprotonation as a consequence of the more acidic local environment. In fact, it is well-known that polyanionic surfaces and polymers determine the formation of acidic local environments by attracting protons from the bulk solution [39,40]. Further information was obtained from analysis of the emission spectra. Once more, the emission spectrum in the presence of POPC/POPG liposomes was very different from the other two spectra, showing the presence of a large shoulder at 425–430 nm (Figure 2). As mentioned above, this blue emission is characteristic of the phenolic form of Luc and can only be observed when Luc is in an environment with very low water content, a condition able to suppress the photoinduced dissociation. Therefore, the presence of a blue shoulder in the emission spectrum of Luc-ApoB_L_ is a clear indication that the Luc moiety is deeply embedded into the POPC/POPG bilayer. This is further confirmed by the 16–18 nm blue shift (from 539 to about 522 nm) of the peak in the green region (Table 1). The analysis of the emission spectra of aLuc-ApoB_L_ is simpler but not less informative. This peptide shows a considerable increase in the fluorescence emission and a 27–28 nm blue shift (from 525 to about 497 nm) only in the presence of POPC/POPG liposomes (Figure 2 and Table 1). Given the solvatochromic nature of aLuc, this is an indication that, only in the case of negatively charged liposomes, the probe is embedded in a hydrophobic environment. Therefore, three different and independent phenomena confirm binding and embedding of labeled ApoB_L_ into the POPC/POPG bilayer: (i) inhibition of the deprotonation of Luc; (ii) inhibition of the photoinduced dissociation of Luc; (iii) the blue shift of Luc and aLuc emission peaks. In this regard, it is worth noting that the interaction of ApoB_L_ with anionic liposomes of phosphatidylcholine and phosphatidylglycerol has been recently demonstrated by using differential scanning calorimetry [41].

Labeled p53pAnt showed a behavior essentially similar to labeled ApoB_L_ (Figure 2), whereas GKY20 showed that it can interact with both types of liposomes. Indeed, the emission spectra of Luc and aLuc-GKY20 and the excitation spectra of Luc-GKY20 in the presence of POPC and POPC/POPG liposomes are very similar (Figure 2). All the emission spectra show a large blue shift compared to those recorded in NaP (Table 1). Furthermore, the emission spectra of Luc-GKY20 show a blue shoulder, thus indicating that the (a)Luc moiety is embedded in a less polar environment. The spectra of PyMPO-(C)GKY20 in the presence of liposomes were found to be very similar to those of aLuc-GKY20 (Figure S7), thus demonstrating that the binding to both liposome types is not an artifact due to (a)Luc. These findings agree with previous studies performed using unlabeled GKY20 and the same model membranes [42]. We do not have a straightforward explanation for the observed varying behavior of the two CAMPs. However, very interestingly, further analyses conducted on labeled GKY20 and ApoB_L_ evidence several additional differences, as described in the next sections.

### 2.6. Interaction of Labeled CAMPs with SDS and LPS Micelles

**Figure 3:**
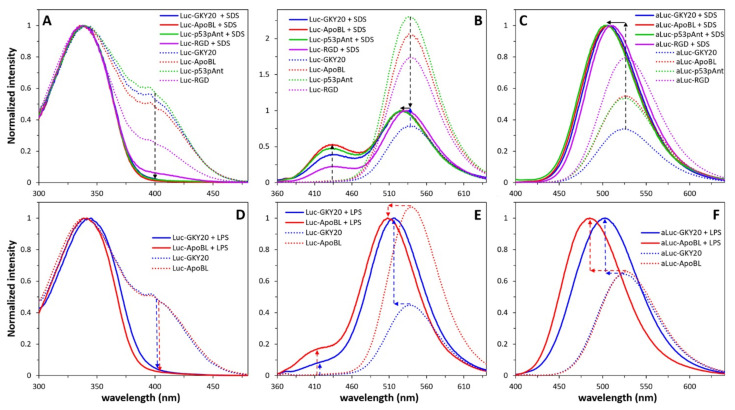
Fluorescence of labeled peptides in the presence of SDS or LPS micelles. (**A**,**D**) Excitation spectra of Luc-labeled peptides (em. = 539; Luc-GKY20 + LPS, em. = 516 nm; Luc-ApoBL + LPS, em. = 508 nm). (**B**,**E**) Emission spectra of Luc-labeled peptides (ex. = 330 nm). (**C**,**F**) Emission spectra of aLuc-labeled peptides (ex. = 363 nm). Solid line, spectra recorded in the presence of SDS or LPS; dotted line, spectra recorded in NaP. In (**B**,**C**,**E**,**F**), spectra recorded in NaP were normalized to the corresponding spectra recorded in the presence of SDS or LPS. Arrows highlight the main changes with respect to NaP.

In order to further confirm the ability of Luc probes to reveal the interaction of peptides with lipidic structures, we also recorded fluorescence spectra in the presence of 25 mM SDS (Figure 3A–C). At this concentration, well above the critical micelle concentration (CMC) of about 8 mM, SDS forms micelles containing an average of 60 molecules [43]. For this reason, SDS has been frequently used as a membrane mimetic, such as for determination of the NMR structure of membrane-binding peptides. However, it should be remembered that SDS is a strong detergent able to interact unspecifically with peptides. Accordingly, in the presence of SDS, not only labeled GKY20, ApoB_L_ and p53pAnt but also labeled RGD showed spectral behavior, suggesting interaction with micelles (Figure 3A–C). Nonetheless, the spectra of labeled RGD were not completely superimposable to those of the other three peptides. For example, in the excitation spectra of Luc-RGD in the presence of SDS, a tail at 400–420 nm is still visible, indicating that not all of the peptide is strongly associated to the micelles (Figure 3A–C). In the emission spectrum of Luc-RGD, the blue peak is less evident than in the corresponding spectra of the other three peptides (Figure 3A–C), and the ratio between the area of the peaks in the blue (380–465 nm) and green (466–640 nm) regions was 0.16, whereas the same ratio for Luc-GKY20, Luc-ApoB_L_ and Luc-p53pAnt was 0.27, 0.36 and 0.33, respectively. It is also evident that the peak in the green region is less blue-shifted than in the case of Luc-GKY20, Luc-ApoB_L_ and Luc-p53pAnt (Figure 3A–C and Table 1).

As in the case of the emission spectra recorded in the presence of liposomes, the spectra of aLuc-labeled peptides recorded in the presence of SDS also showed a significant increase in emission intensity, as well as a blue shift. However, again, the blue shift was smaller in the case of aLuc-RGD (Figure 3A–C and Table 1). These results indicate that the hydrophilic RGD peptide interacts less tightly with the SDS micelles than Luc-GKY20, Luc-ApoB_L_ and Luc-p53pAnt.

GKY20 and ApoB_L_ bind to and neutralize lipopolysaccharides (LPS), the main components of Gram-negative bacteria outer membrane, with strong proinflammatory effects in higher eukaryotes [19,20,44]. These very complex molecules have three portions with different compositions: (i) lipid A, a phosphorylated disaccharide bearing 4–6 fatty acid residues; (ii) the core, an oligosaccharide with a variable number of phosphate groups; (iii) the O-antigen, a species and strain-specific polysaccharide [45]. It should be noted that pure LPS, like SDS, does not form regular bilayers but negatively charged micelles [46]. However, the CMC of LPS is usually in the low micromolar range [46]. The ability to bind and neutralize the strong pro-inflammatory and sometimes life-threatening effects of LPS is one of the most pharmacologically relevant properties of CAMPs [45]. Therefore, the study of the CAMP/LPS interaction is of outstanding importance.

Accordingly, we studied the interaction of labeled GKY20 and ApoB_L_ by using a commercially available LPS from *E. coli* strain 0111:B4. Firstly, we recorded the excitation and the emission spectra of Luc-GKY20 and Luc-ApoB_L_ in the presence of 200 µg/mL LPS, i.e., a concentration well above the CMC of this LPS (about 1.3–1.6 µM corresponding to 13–16 µg/mL) [46]. As expected, the binding of the two peptides to LPS caused the disappearance of the shoulder at 400 nm in the excitation spectra (Figure 3D–F). The emission spectra of Luc-GKY20 and Luc-ApoB_L_ show the expected blue shift of the peak in the green region—even larger than those observed in the presence of SDS and liposomes (Table 1) However, surprisingly, only a very low shoulder was observed in the blue region, especially in the case of Luc-GKY20 (Figure 3D–F). The ratio between the area of the peaks in the blue (365–435 nm) and green (436–640 nm) regions is 0.046, and 0.095 for Luc-GKY20 and Luc-ApoB_L_, respectively, i.e., even lower than the ratio observed for the Luc-RGD peptide in the presence of SDS. Very interestingly, the peak in the blue region is also blue-shifted (about 20 nm) with respect to the same peak observed in the case of liposomes and SDS. Finally, the emission spectra of aLuc-GKY20 and aLuc-ApoB_L_ show, as expected, an increase in emission intensity and a large blue shift, particularly in the case of aLuc-ApoB_L_ (41 nm). These findings suggest that the orientation of the Luc moiety when the peptides are bound to LPS is very different from that adopted when the peptides are bound to liposomes and SDS micelles. The high blue-shift values suggest that the (a)Luc moiety is in a very non-polar environment, while the very weak emission in the blue region indicate that the phenolic OH group of Luc points toward the solvent or a proton acceptor in the LPS (e.g., a basic group in the lipid A of LPS). Possible orientations of the Luc moiety in LPS and SDS or liposomes explaining the observed variations in excitation and emission spectra are schematically drawn in Figure S8.

### 2.7. Interaction of Labeled CAMPs with Non-Micellar LPS

**Figure 4:**
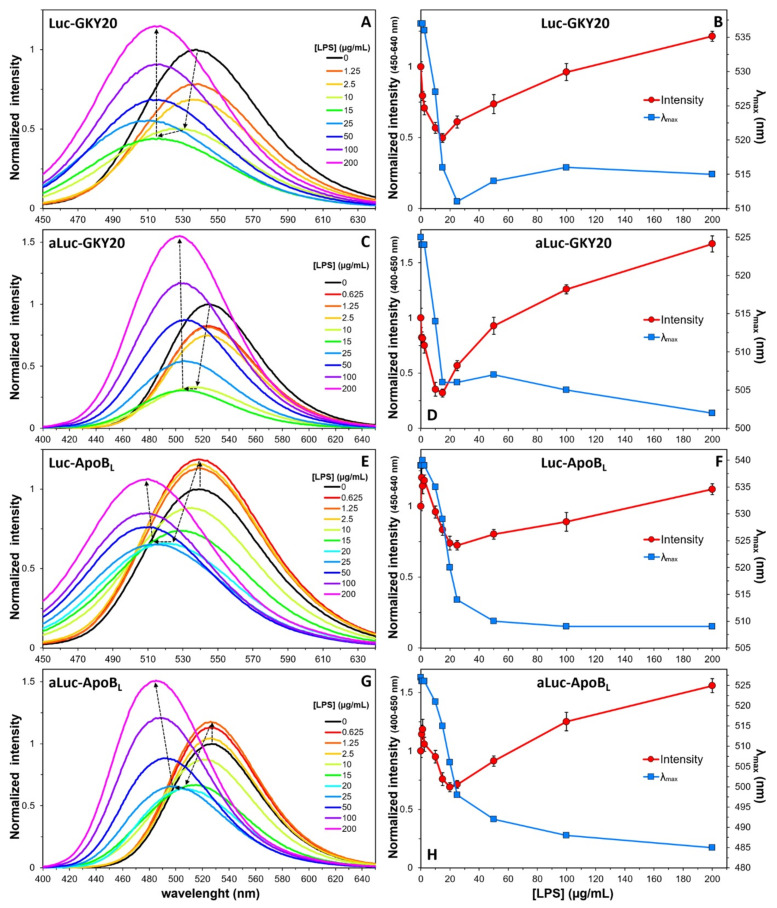
Fluorescence of the labeled peptides in the presence of increasing concentrations of LPS (0–200 µg/mL). (**A**,**C**,**E**,**G**) Emission spectra of the peptides recorded after excitation at 330 nm (Luc-labelled peptides) and 363 nm (aLuc-labelled peptides). Spectra recorded in the presence of LPS were normalized to the corresponding spectra recorded in NaP (black lines). Arrows highlight the main changes with respect to NaP. (**B**,**D**,**F**,**H**) Variation of total fluorescence (in the indicated ranges) and of the λ_max_ values as a function of LPS concentration.

Next, we recorded the emission spectra of Luc-GKY20, Luc-ApoB_L_, aLuc-GKY20 and aLuc-ApoB_L_ at a constant peptide concentration in the presence of increasing concentrations of LPS (Figure 4). All peptides showed a turn-off of the fluorescence associated with a considerable blue shift of λ_max_ values for LPS concentrations up to 10–20 µg/mL, followed by a turn-on phase with smaller changes in λ_max_ values. The biphasic nature of the process is clearly visible by plotting the area beneath the spectra and the λ_max_ values as a function of the LPS concentration (Figure 4B,D,F,H). Only in the case of (a)Luc-ApoB_L_, a turn-on phase with no shift in λ_max_ values was visible at very low LPS concentrations (0–2 µg/mL).

A similar behavior has been previously described for GKY25, HVF18 and VFR12, three CAMPs derived, like GKY20, from the C-terminus of human thrombin. In particular, GKY25 is a variant of GKY20, with five additional residues at the C-terminus [47]. In that case, the authors, exploiting the intrinsic fluorescence of the single tryptophan residue present in all the thrombin-derived CAMPs, observed a biphasic process with a turn-off phase for LPS concentrations below 10 µg/mL and a turn-on phase at higher concentrations. Therefore, the turn-off/turn-on switch seems to be independent of the nature and position of the fluorophore. As the CMC of *E. coli* LPS is about 16 µg/mL, it can be speculated that the turn-off and turn-on phases might be the result of the binding to free and micellar LPS, respectively. The additional turn-on phase observed at very low LPS concentrations only in the case of (a)Luc-ApoB_L_ might be due to a conformational change in this peptide induced by the presence of small amounts of LPS. In this regard, it is worth noting that the CD spectra of unlabeled GKY20 and ApoB_L_ in the presence of LPS are quite different [19,20]. In the case of ApoB_L_, circular dichroism studies suggest that this peptide adopts a β-sheet conformation upon interaction with LPS [20]. On the other hand, the CD spectrum of GKY20 in the presence of LPS is not similar to any of the CD spectra of model conformations [19]. It could also be hypothesized that LPS might form small aggregates even below the CMC, which could be responsible for the turn-off phase, whereas the first turn-on phase observed at very low LPS concentrations would be due to the association of (a)Luc-ApoB_L_ with truly monomeric LPS molecules. This aspect would require further investigation, which lies outside the scope of this work.

**Figure 5:**
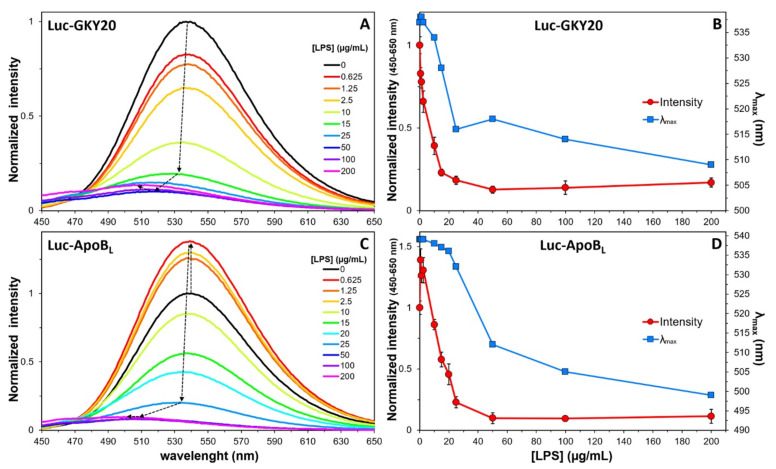
Fluorescence of the Luc-labeled peptides in the presence of increasing concentrations of LPS (0–200 µg/mL). (**A**,**C**) Emission spectra of Luc-labeled peptides recorded after excitation at 400 nm. Spectra recorded in the presence of LPS were normalized to the corresponding spectra recorded in NaP. Arrows highlight the main changes with respect to NaP. (**B**,**D**) Variation of total fluorescence (in the indicated ranges) and of the λ_max_ values as a function of LPS concentration.

We also recorded the emission spectra of Luc-GKY20 and Luc-ApoB_L_ after excitation at 400 nm in order to follow the binding process by monitoring the disappearance of the phenolate form in the solution (Figure 5). As expected for a saturable binding process, we observed a progressive reduction in the fluorescence, which reached a minimum at about 50 µg/mL LPS for both peptides.

### 2.8. Quantitative Analysis of the Peptide/LPS Interaction

**Figure 6:**
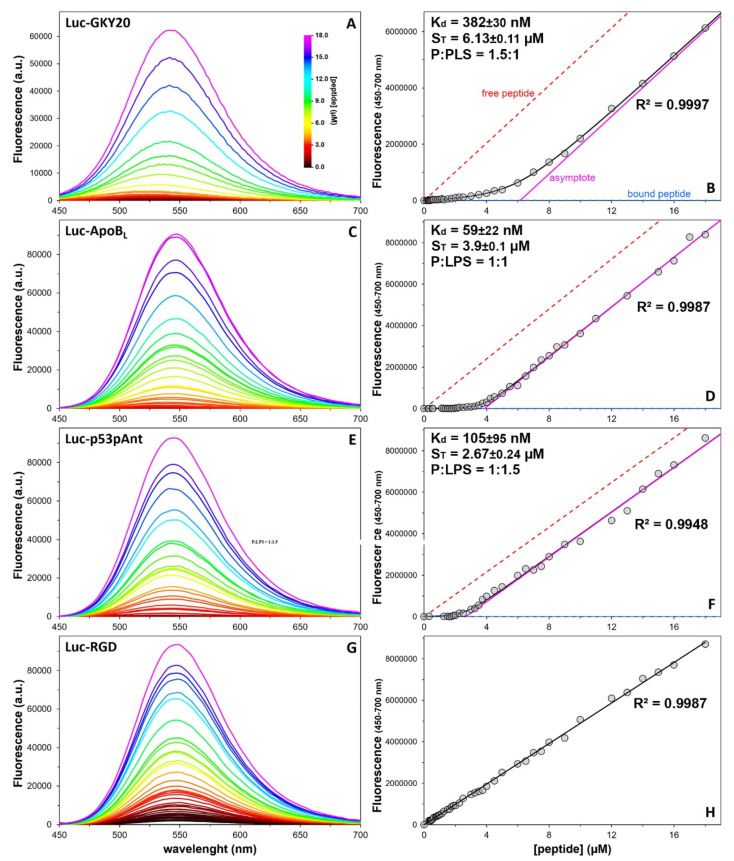
Determination of the K_d_ values and stoichiometry for the peptide/micellar LPS interaction. (**A**,**C**,**E**,**G**) Emission spectra of Luc-labeled peptides (0–18 µM) in the presence of 40 µg/mL LPS (ex. = 400, 415, 425 and 410 nm, respectively). (**B**,**D**,**F**,**H**) Variation of total fluorescence (450–700 nm) as a function of peptide concentration. The dashed lines are the expected fluorescence of the free and bound peptide, respectively. Black lines, K_d_ and stoichiometry (S_T_) values were obtained using the equation described in Supplementary Materials. The ratio P:LPS was calculated from S_T_, assuming that *E. coli* LPS has an average molecular weight of 10 kDa. In (**H**), data were fitted to a straight line.

The curves measured at constant peptide concentration and variable LPS concentration could be used to determine K_d_ values (Supplementary Materials, Section S9). However, as such K_d_ values would be the result of measurements obtained using concentrations below and above the CMC of LPS, their meaning would be questionable. In order to determine the K_d_ value of Luc-labeled peptides for micellar LPS, we repeated the experiment at variable Luc-labeled peptide concentration and constant LPS concentration (40 µg/mL corresponding to ~4 µM). The experimental data were fitted to the model described in Section S9, Supplementary Materials. The model allows for the estimation not only the K_d_ value but also the number of binding sites and hence the stoichiometry of binding. The spectra and the fittings are shown in Figure 6. Luc-GKY20, Luc-ApoB_L_ and Luc-p53pAnt show K_d_ values in the range 50–400 nM. As expected, Luc-RGD did not bind to LPS, and the resulting plot of the fluorescence emission as function of the peptide concentration was a straight line (Figure 6). The fact that the anticancer peptide Luc-p53pAnt binds to LPS with affinity comparable to those of the two CAMPs is not surprising, considering that it has an amino-acid composition similar to that of the CAMPs (Figure S1) and that it is the most cationic of the three peptides (Table 3).

On the other hand, the three peptides show very different binding stoichiometries (Figure 6). Only Luc-ApoB_L_ binds to LPS in a 1:1 ratio. In the case of Luc-GKY20, three molecules of the peptide bind to two molecules of LPS, whereas in the case of Luc-p53pAnt, two molecules of the peptide bind to three molecules of LPS. The higher number of binding sites found for Luc-GKY20 might be due to the fact that this peptide is considerably shorter than the other two. However, Luc-ApoB_L_ and Luc-p53pAnt have exactly the same length; therefore, the different stoichiometry might be due to a different mode of binding or a different fold adopted by the peptides upon binding.

### 2.9. Interaction of Labeled CAMPs with E. coli Cells

In order to study the interaction of GKY20 and ApoB_L_ with whole bacterial cells, we recorded the emission spectra of the labeled peptides in the presence of *E. coli* cells at an optical density of 0.1 OD_600_ (corresponding to about 0.63 × 10^9^ CFU/mL). The emission spectra of Luc-GKY20 and Luc-ApoB_L_ obtained after excitation at 330 nm show that in both cases, binding to *E. coli* cells causes a moderate decrease in the fluorescence emission (Figure S9A), accompanied by a blue shift of 16 nm in the case of Luc-GKY20 (a value slightly lower than those observed in the case of liposomes and micellar LPS) and of only 5 nm in the case of Luc-ApoB_L_ (Table 1). In the blue region, the emission of Luc-GKY20 was higher than that of Luc-ApoB_L_, which is the opposite of what was observed in the case of LPS (Figure S9B). The differences between the spectra of the labeled peptides in the presence of whole bacterial cells and those obtained in the presence of liposomes and purified LPS might be due to the fact that the outer membrane of Gram-negative bacteria is a very complex mixture of LPS, phospholipids and proteins.

The emission spectra of Luc-GKY20 and Luc-ApoB_L_ obtained after excitation at 400 nm show a strong decrease in fluorescence emission (Figure S9C), likely due to a reduced hydrolysis of the Luc hydroxyl group of the cell-bound peptides.

Finally, *E. coli* cells caused a significant increase in the fluorescence emission of aLuc-GKY20 and a slight decrease in the fluorescence emission of aLuc-ApoB_L_ (Figure S9D). In the case of aLuc-labeled peptides, we also observed a blue shift lower than that observed in the case of liposomes and micellar LPS (Table 1).

The spectra shown in Figure S9 were recorded after an incubation time of 20 min in the case of labeled GKY20 and of 120 min in the case of labeled ApoB_L_. The different incubation times were necessary for an unexpected difference in the binding kinetic of the two peptides, as shown in Figure S9E. In the case of Luc-GKY20, the slope of the curve obtained by plotting fluorescence intensity (ex. = 400 nm; em. = 539 nm) as a function of time was about 6.7 times higher than that observed in the case of Luc-ApoB_L_. Intriguingly, in the case of purified LPS, the binding process was complete within the preparation time of the samples (about 60 s) for both peptides. The reasons for such differences were not further investigated. Nonetheless, these results highlight another useful application of Luc labeling.

We also observed *E. coli* cells treated with the labeled peptides (3 µM) using a fluorescence microscope equipped with a mercury arc lamp (Figures S10 and S11). In the case of GKY20-treated cells, in addition to homogeneously labeled cells, we observed several cells with a heterogeneous labeling pattern (Figures S10K,N and S11E). The same pattern was observed in *E. coli* cells treated with PYMPO-(C)GKY20, indicating that heterogeneous labeling is not an artifact of Luc labeling (Figure S10A–D). For incubation times longer than 30 min, we observed an increased amount of highly fluorescent and large bodies (Figure S11), likely aggregates of cell debris and/or dead cells. This is not surprising, as GKY20 and ApoB_L_, like many CAMPs, cause cell lysis [19,44].

### 2.10. Confocal Laser Scanning Microscopy

Confocal Laser Scanning Microscopy (CLSM) allows for the attainment of high resolution tridimensional images of biological samples by stacking several bidimensional images taken at different depths [48]. As in CLSM, fluorophore excitation is obtained through high-power but very narrow laser beams. It is mandatory that the excitation peak of the chosen fluorescent label overlaps the wavelength of one of the available laser lines [48]. Moreover, the fluorescent label should be photostable enough to avoid a quick bleaching of the sample. This is especially important in the case of live-imaging applications. We have already shown that Luc and aLuc are very stable fluorophores. As regards the excitation wavelength, it is interesting to note that the excitation spectra of neutral Luc and its phenolate show an isosbestic point at about 355 nm, a wavelength very close to that of the argon-ion laser (351 nm). Therefore, this laser could be used to simultaneously excite both forms of Luc. A blue diode laser (405 nm) could be used to excite aLuc, the broad excitation peak of which is centered at 360–370 nm but has also a remarkable tail in the violet region. Differently from an argon-ion laser, which is not common, the blue diode laser is present on most CLSM devices used to excite blue fluorophores. In order to evaluate the suitability of aLuc as label for CLSM, we incubated HaCaT and HeLa cells with aLuc-p53pAnt or aLuc-RGD for 60–150 min; hence, samples were either observed or incubated for 15 additional min with LysoTracker™ Red DND-99, a red probe that is actively taken up by acidic organelles [49], and/or NucRed™ Live 647, a far-red cell-permeant vital stain for nucleic acids [50,51]. Samples were observed without any further wash or treatment in order to minimize artifacts and to mimic the conditions of live imaging.

Non-cell-specific uptake of p53pAnt has been previously demonstrated by Western blotting of cell lysates and immunostaining by an antibody specific to the p53-derived portion of p53pAnt [25,52], whereas confocal microscopy with a rhodamine B-labeled peptide showed that it accumulates both in the cytosol and the nuclei of two prostate cancer cell lines [52]. Our CLSM analysis of HaCaT and HeLa cells treated with aLuc-p53pAnt confirm the previous findings. In the case of HaCaT cells treated with aLuc-p53pAnt, the peptide was localized at the cell periphery, mainly in the form of circular spots, thus suggesting the presence of the peptide in an endosomal compartment (Figure S12). A very small fraction of the cells, however, displayed a very strong signal, partly diffused into the cell and partly associated to the nucleus (Figure S13). The signal was very strong at the nuclear periphery but also present inside the nucleus in the form of one or more patches of different dimensions (Figure S13). As discussed below, colocalization studies with NucRed Live confirm that the peptide binds to nucleic acids. Very interestingly, in the case of HeLa cells, the proportion between the two types of staining pattern was reversed, with the highly stained cells being predominant (Figure S13). Considering that p53pAnt is much more toxic for HeLa cancer cells than for HaCaT cells (Figure S4) and that it has been suggested that p53pAnt induces apoptosis, it can be speculated that highly stained cells are apoptotic or pre-apoptotic cells. It is well known that apoptosis causes relevant alterations of cell membranes, e.g., externalization of phosphatidylserine [53], a negatively charged lipid, which could determine an increased afflux into the cytosol of p53pAnt. Once in the cytosol, p53pAnt could migrate into the nucleus and bind nucleic acids due to its high positive charge.

**Figure 7:**
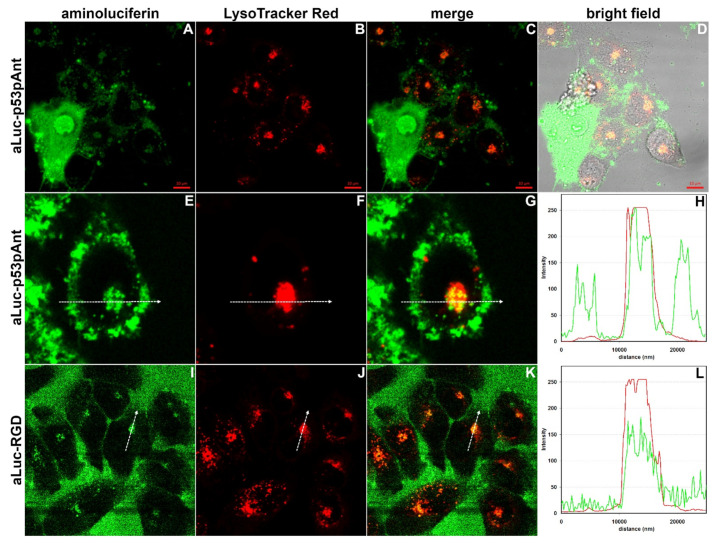
CLSM images of HaCaT cells treated with aLuc-p53pAnt or aLuc-RGD. (**A**–**D**) Cells were incubated with aLuc-p53pAnt for 120 min and with LysoTracker™ Red DND-99 for 15 additional minutes. Bar = 10 µm. (**E**,**F**) Single representative HaCaT cell treated with aLuc-p53pAnt (from Figure S14A–D). White arrow = 25 µm. (**I**–**K**) Cells were incubated with aLuc-RGD for 90 min and with LysoTracker™ Red DND-99 for 15 additional minutes (bright field in Figure S16E). White arrow = 25 µm. (**H**,**L**) Fluorescence intensity across the white arrows in panels (**E**–**G**) and (**I**–**K**), respectively (green curve, aLuc; red curve, LysoTracker Red).

HaCaT cells treated with aLuc-p53pAnt and LysoTracker Red showed, as expected, numerous red spots (Figure 7 and Figure S15). Very interestingly, we observed partial colocalization between LysoTracker Red and aLuc-p53pAnt (Figure 7E,F and Figure S14A–D). Cells showing a strong aLuc-p53pAnt fluorescence did not show the presence of LysoTracker Red (Figure 7A–D). The same pattern was observed in the case of HeLa cells, except that, again, the frequency of cells showing only the strong fluorescence of aLuc-p53pAnt was higher than in the case of HaCaT cells (Figure S15A–D). Accumulation of LysoTracker Red, requiring acidification of endosomes, is expected only in metabolically active cells; therefore, these findings are in agreement with the hypothesis that cells strongly stained with aLuc-p53pAnt are apoptotic or pre-apoptotic cells.

Unexpectedly, our attempts to perform three-color imaging by staining aLuc-p53pAnt-treated HaCaT cells with LysoTracker Red and NucRed Live for 15 min revealed an alteration of the localization both of LysoTracker Red and of aLuc-p53pAnt (Figure S14E–H). The change was particularly evident in the case of LysoTracker Red, which appeared more homogeneously diffused inside the cell than in the absence of NucRed. On the contrary, the signal of aLuc-p53pAnt appeared less diffused and with more defined spots (Figure S14E). The alteration in the distribution of aLuc-p53pAnt was more pronounced by co-incubating HaCaT cells with NucRed Live and aLuc-p53pAnt for one hour (Figure S14I–L). In that case, in the majority of the cells, aLuc-p53pAnt appeared as numerous large and well-defined spots at the cell periphery. In the case of aLuc-p53pAnt-treated HeLa cells, staining with NucRed Live for 15 min had minor effects on the appearance of the putative apoptotic/pre-apoptotic cells (Figure S15E–H). Interestingly, inside the nuclei of these cells, aLuc-p53pAnt and NucRed Live were essentially colocalized (Figure S15M–O), thus suggesting that aLuc-p53pAnt is bound to nucleic acids. Similarly to what was observed in the case of HaCaT cells, LysoTracker Red was more diffused in the presence of NucRed Live (Figure S15). When HeLa cells were treated with NucRed Live for one hour, we no longer observed the putative apoptotic/pre-apoptotic cells (Figure S15I–L), and the staining pattern was very similar to that of the HaCaT cells treated for one hour with NucRed Live (Figure S14I–L). Very interestingly, NucRed Live also proved to change the behavior of aLuc-RGD, as described below.

RGD is a known integrin ligand derived from fibronectin; therefore, it is expected to undergo receptor-mediated endocytosis, as demonstrated for other similar peptides [54]. In particular, it has been demonstrated that peptide GRGDNP, which differs by one amino acid (N at position 5) from RGD, is endocytosed in Molt-4 cells (leukaemic T-cell line) [55]. Differently from aLuc-p53pAnt, aLuc-RGD was homogeneously dispersed in the culture medium in the case of both HaCaT and HeLa cells, which appeared as non-fluorescent regions (Figure 7I–L and Figure S16, respectively). Nonetheless, as expected, an intracellular signal was visible and colocalized with LysoTracker Red (Figure 7I–L and Figure S16). In the presence of NucRed Live, aLuc-RGD was no longer visible inside the cells, whereas the signal of LysoTracker Red appeared homogeneously diffused inside the cytosol (Figure S16), as observed in the case of the analysis of aLuc-p53pAnt.

Overall, NucRed Live seems to be able to interfere with endocytosis and intracellular trafficking. NucRed Live is a relatively recent stain, and we did not find other reports regarding its potential biological effects. On the other hand, it has been demonstrated that another far-red, membrane-permeable nuclear stain, DRAQ5™, alters membrane fluidity and inhibits the internalization of bacterial toxins [56]. Reduction in membrane fluidity and/or alteration of membrane potential by NucRed Live might prevent translocation of p53pAnt from the endosomes to cytosol, thus causing accumulation of the peptide in the lumen of endosomes. This, in turn, would prevent its interaction with p53, thus inhibiting the proapoptotic effect of the peptide. The effect on LysoTracker Red could be explained, at least in part, by assuming that NucRed Live is also able to inhibit endosome acidification. We did not further study the effects of NucRed Live, as this is beyond the scope of the current work; nonetheless, the analysis reported herein clearly shows that aLuc is very well suited as a probe for CLSM.

## 3. Discussion

We have shown that Luc and aLuc are very well suited as environment-sensitive fluorescent labels for peptides. Labeling is fast, quantitative, very specific and can be performed in very mild conditions (RT, buffer phosphate at pH 7–7.5) using commercially available reagents and peptides with an N-terminal cysteine that can be prepared either by chemical synthesis or by several recombinant strategies ([32] and references therein). Luc and aLuc are photostable fluorophores with a large Stoke shift (about 210 and 145 nm, respectively) and a high quantum yield. Moreover, they are small molecules (low molecular weight and low solvent-accessible surface), uncharged (aLuc) or with a low percentage of ionized form at pH 7 (Luc), neither highly polar nor particularly hydrophobic, thus minimizing the impact of labeling on the structure of the peptides and presumably on their properties. Furthermore, N-terminal labeling is very well suited for most peptides, and indeed, it is a very common choice. On the other hand, Luc and aLuc possess complementary properties. Luc has a very uncommon dual emission with a main emission in the green region (~539 nm), commonly observed in aqueous buffers and a blue emission (~450 nm), which can only be observed in an environment with a very low water content. Moreover, both peaks undergo significant blue shifts (20–30 nm) in hydrophobic environments. This behavior makes Luc a very useful probe for the study of the interactions of peptides with membranes, liposomes, micelles of detergents and LPS. The very large difference between the excitation maxima of the neutral and the phenolate form (330 and 400 nm, respectively) makes Luc an intriguing probe for the pH range of 6.5–9.5 (hypothesizing pK_a_ values in the range 7.5–8.5 for peptide-bound Luc). Moreover, it can be exploited to detect binding events that influence its ionization state, as shown in the case of binding to liposomes, SDS, LPS and *E. coli* cells, and to determine K_d_ values and stoichiometries. aLuc is not sensitive to pH and shows less pronounced variations in fluorescence emission. However, it is, in turn, a strongly solvatochromic fluorophore, showing blue shifts up to 40 nm. This makes aLuc an alternative probe for the study of the interactions of peptides with their targets. In addition, aLuc is very well suited as a label for CLSM, and it does not require special equipment. It can be efficiently excited by the common 404 nm laser, and emitting at 500–520 nm, it allows for colocalization studies with the many commercially available and widely used orange, red and far-red probes, as was shown by our analysis of the colocalization of p53pAnt and RGD with LysoTracker™ Red DND-99 and NucRed™ Live 647.

It is worth noting that we have only explored a minimal part of the potentialities of Luc and aLuc as fluorescent labels for peptides. For example, when N-terminal labeling is not suitable or when large proteins have to be labeled, a 1,2-aminothiol functionality could be introduced at internal positions, e.g., as N^ε^-cysteinyl-L-lysine [57], or by modifying a cysteine residue [58]. Furthermore, Luc and aLuc might be bound to internal cysteine or lysine residues via the carboxyl group of luciferins, exploiting conventional chemical strategies to crosslink carboxyl groups to amines and thiols [59]. Even more interestingly, in the attempt to find new substrates for firefly luciferase, an astonishing number of Luc and, in particular, aLuc derivatives and analogues have been published [60,61]. Many of these compounds are fluorescent and show intriguing properties; for example, many aLuc derivatives show red-shifted λ_max_ values (up to 576 nm) and/or altered solvatochromic behavior (increased or decreased, depending on the nature of the substituents bound to the N6 nitrogen atom) [11,61,62]. Moreover, halogenated luciferins show decreased pK_a_ values—e.g., 7-F- and 7-Cl-luciferin have pK_a_ values of 7.1 and 6.7, respectively [63]—thus expanding the useful pH range in applications based on pH-dependent fluorescence. Most of the cited derivatives and analogues were synthetized by reacting the corresponding 2-cyano-benzothiazole with cysteine. Thus, they could be directly generated at the N-terminus of peptides with a terminal cysteine residue. The others might be linked to peptides through the activation of their carboxylate group, which, being essential for the catalytic activity of firefly luciferase, is present in all the analogues.

Therefore, Luc and aLuc can reasonably be regarded as the prototypes of a huge new and variegated family of fluorescent labels for proteins and peptides.

## 4. Materials and Methods

### 4.1. Materials and General Methods

Materials and general methods can be found in Section S1 (Supplementary Materials). The sequences of the peptides GKY20, ApoBL, p53pAnt and RGD are shown in Figure S1, and their preparation, purification and labeling are described in Sections S2–S6 (Supplementary Materials).

### 4.2. Steady-State Fluorescence Spectroscopy in Water/Organic Solvent Mixtures and SDS

Fluorescence spectra were recorded on a Fluoromax-4 fluorometer (Horiba, Edison, NJ, USA) using a 1 cm path length quartz cuvette at a temperature of 25 °C, using a peltier that can ensure an accuracy of ±0.1 °C. All experiments were carried out at a fixed peptide concentration (2 µM) in 10 mM sodium phosphate (NaP), pH 7.4, at 25 °C, unless otherwise stated. The excitation wavelengths were set to 330 nm (Luc-labeled peptides, phenol form), 400 nm (Luc-labeled peptides, phenolate form), 363 nm (aLuc-labeled peptides), 330 nm (mLuc-GKY20) and 408 nm for 1-[2-(maleimido)ethyl]-4-[5-(4-methoxyphenyl)-2-oxazolyl]pyridinium-labeled GKY20 [PyMPO-(C)GKY20]. The excitation spectra were recorded by varying the wavelength of excitation between 200 nm and 500 nm (em. = 539 nm). To evaluate the solvatochromic properties of labeled peptides, fluorescence spectra were recorded in the presence of NaP:methanol (50% *v*:*v*), NaP:isopropanol (50% *v*:*v*) and SDS (25 mM) under the experimental condition described above.

### 4.3. Steady-State Fluorescence Spectroscopy in the Presence of Liposomes

Liposome preparation is described in Section S8, Supplementary Materials. Fluorescence spectra were recorded on a FluoroMax-4 fluorometer (Horiba, Kyoto, Japan). The emission spectra of Luc-labeled peptides were acquired, upon excitation at 330 nm, in the range 350–650 nm. For the aLuc-labeled peptides, the emission spectra were acquired in the range 380–700 nm, upon excitation at 363 nm. In addition, for Luc-labeled peptides, excitation spectra were also recorded. The excitation spectra were recorded by varying the wavelength of excitation between 275 nm and 480 nm and monitoring the emission at 539 nm. All the spectra were recorded at a lipid-to-peptide ratio of 200 in 10 mM NaP, pH 7.4. The concentration of peptides was in the range of 2.2–3.6 μM.

### 4.4. Quantum-Yield Determination

Fluorescence quantum yields were determined for all the labeled peptides in 10 mM phosphate buffer, pH 7.4, in a mixture composed of phosphate buffer and isopropanol in the ratio 1:1 (*v*:*v*) and in acetate buffer, pH 5. Determination of the quantum yields was performed by comparing the fluorescence of samples to that of a standard, as previously described [64]. Fluorescein (in 0.1 M NaOH) was used as standard in the case of (a)Luc- and PyMPO-labeled peptides, whereas coumarin-6 (in pure ethanol) was used in the case of mLuc-labeled GKY20.

### 4.5. pK_a_ Determination of Luc-Labeled Peptides

In order to measure pK_a_ values, phenolate concentration was evaluated by titrating a solution of Luc-labeled peptide (2 μM) as a function of pH (0.2 M sodium acetate, pH 4–6; 0.2 M sodium phosphate, pH 6–7.4; 0.2 M Tris/HCl, pH 7–9; 0.2 M Glycine/NaOH, pH 8–11). The excitation wavelength was set to 400 nm. Excitation spectra were recorded at 539 nm. pK_a_ values were determined by GraphPad Prism software (version 6, San Diego, CA, USA) by plotting variation of total fluorescence (450–700 nm) as a function of pH values.

### 4.6. Interaction of Labeled CAMPs with LPS

Binding of CAMPs (2 μM) to LPS (200 μg/mL) from *E. coli* 0111:B4 (MW 10,000) [46] was performed in 10 mM NaP buffer, pH 7.4. Mixtures were equilibrated at 25 °C for 10 min before recording emission (Luc-peptides, ex. = 330; aLuc-peptides, ex. = 363 nm) and excitation spectra (Luc-GKY20, em. = 516 nm; Luc-ApoB_L_, em. = 508 nm) by means of a FluoroMax-4 fluorimeter. To test the influence of micellar and sub-micellar LPS concentration on CAMP fluorescence (2 μM), emission spectra (Luc-peptides, ex. = 330 or 400 nm; aLuc-peptides, ex. = 363 nm) were also recorded in the presence of increasing concentrations of LPS (0.62 e 200 μg/mL). Variation of total fluorescence (450–700 nm) was reported as a function of LPS concentration. The assays to determine K_d_ and binding stoichiometry of Luc-peptides toward LPS were carried out in 96-well polystyrene microtiter plates containing 100 μL of peptide/LPS mixtures. Spectra were recorded using a Synergy^TM^ H4 microplate reader (BioTek Instruments Inc., Winooski, VT, USA) in 10 mM NaP buffer, pH 7.4, in the presence of 40 μg/mL (≈4 μM; MW 10,000) LPS and Luc-peptides (0.25–18 μM). Mixtures were incubated 15 min before emission spectra were recorded by excitation between 400 and 425 nm (phenolate form). Variation of total fluorescence (450–700 nm) was reported as a function of peptide concentration, and data were fitted to the model using Graphpad Prism (Supplementary Materials, Section S9).

### 4.7. Interaction of Labeled CAMPs with E. coli Cells

Bacterial *E. coli* ATCC 25922 strain was cultured in LB medium at 37 °C overnight. Culture was diluted 1:100 in fresh LB medium, and bacteria were grown until 1 OD_600_ optical density. Cells were collected by centrifugation at 8000× *g* for 5 min at 4 °C, washed three times in 10 mM NaP buffer, pH 7.4, and suspended at 1 OD_600_ concentration (10x cell stock solution) in the same buffer. The bacteria mixture was stored on ice until use. Binding of labeled CAMPs to *E. coli* cells was performed in 10 mM NaP buffer, pH 7.4, in the presence of 0.1 OD_600_ bacterial cells and 2 μM peptides. Mixtures were incubated at 25 °C for 20 min [(a)Luc-GKY20] and 120 min [(a)Luc-ApoB_L_] before recording emission spectra (Luc-peptides, ex. = 330 and 400 nm; aLuc-peptides, ex. = 363 nm).

### 4.8. Kinetic Analysis

Binding kinetic to LPS and *E. coli* cells was carried out in 10 mM NaP buffer, pH 7.4, in the presence of either 50 µg/mL LPS or 0.1 OD_600_ bacterial cells prepared as described above. Binding reactions were started by adding peptides (2 μM) and manually mixing the samples for 40 s. Binding was monitored, exciting at 400 nm and reading at 539 nm. One reading per minute was performed over 16 min observation time. Samples were not irradiated in the period between two readings in order to minimize peptide photobleaching. Photobleaching of peptides was also verified by control experiments carried out in the absence of LPS and cells.

### 4.9. Microscopy Analysis of E. coli Cells Treated with the Labeled Peptides

Binding of labeled CAMPs (3 μM) to *E. coli* cells was performed in 10 mM NaP buffer, pH 7.4, in the presence of 0.1 OD_600_ bacterial cells. Fluorescence microscopy images of treated and untreated *E. coli* cells were taken over 50 min incubation at 25 °C. For this purpose, 10 µL of each sample was observed with an Olympus BX51 fluorescence microscope (Olympus, Tokyo, Japan) using DAPI (aLuc-labeled peptides) and FITC (Luc-labeled peptides) filters. Standard acquisition times were 1000 ms. Images were captured using an Olympus DP70 digital camera. The experiments were performed at least three times.

### 4.10. Interaction of aLuc-p53pAnt and aLuc-RGD with HeLa and HaCaT Cells

Normal human keratinocytes (HaCaT) and human cancer epithelial cells (HeLa cells) were cultured in Dulbecco’s Modified Eagle’s Medium (DMEM), supplemented with 10% fetal bovine serum (FBS), 2 mM L-glutamine and 1% penicillin–streptomycin in a 5% CO_2_ humidified atmosphere at 37ºC. HaCaT and HeLa cells were seeded in chambered well plates (500 μL/well; Nunc™ Lab-Tek™ Chambered Coverglass systems, Thermo Fisher Scientific, Waltham, MA, USA) with a density of 4.5 × 10^4^ and 2.5 × 10^4^/well, respectively, and then grown at 37 °C for 48 h. Cells were washed three times with PBS and then incubated with aLuc-peptides (10 µM) for 1 h in medium without FBS, supplemented with 2 mM L-glutamine and 1% penicillin–streptomycin in a 5% CO_2_ humidified atmosphere at 37 °C. Lysotracker^TM^ Red DND-99 and NucRed™ Live 647 (Thermo Fisher Scientific, Waltham, MA, USA) were added to the cells at the concentrations recommended by the producer and incubated for 15 min in a 5% CO_2_ humidified atmosphere at 37 °C. The samples were then analyzed using a confocal laser scanning microscope (Zeiss LSM 710, Zeiss, Germany) and a 63X objective oil-immersion system. Acquired images were analyzed using the Zen Lite 2.3 software package. In the case of the images shown in Figure 7A–D the intensity of the green channel was increased in order to show both the apoptotic and non-apoptotic cells. Each experiment was performed in triplicate.

## Figures and Tables

**Table 1:** λ_max_ and blue-shift values of the labeled peptides.

Peptide	λ_max_ (nm) ^a^							
	NaP pH 7.4	MeOH 50%	IPA 50%	SDS (25 mM)	POPC +POPG	POPC	LPS (200 µg/mL)	*E. coli* Cells
Luc-GKY20	538	533 (5)	531 (7)	526 (12)	520 (19)	520 (19)	516 (23)	522 (16)
Luc-ApoB_L_	539	533 (6)	533 (6)	526 (13)	522 (17)	539 (0)	508 (31)	534 (5)
Luc-p53pAnt	539	533 (6)	533 (6)	526 (13)	522 (17)	539 (0)	nd ^b^	nd
Luc-RGD	539	533 (6)	533 (6)	533 (6)	539 (0)	539 (0)	nd	nd
aLuc-GKY20	525	515 (10)	510 (15)	506 (19)	499 (26)	499 (26)	503 (22)	509 (16)
aLuc-ApoB_L_	527	516 (11)	512 (15)	505 (22)	497 (27)	527 (0)	486 (41)	518 (9)
aLuc-p53pAnt	526	516 (10)	515 (11)	504 (22)	499 (25)	526 (0)	nd	nd
aLuc-RGD	526	517 (9)	513 (13)	510 (16)	526 (0)	526 (0)	nd	nd
mLuc-GKY20	439	429 (10)	424 (15)	422 (17)	nd	nd	nd	nd
PyMPO-(C)GKY20	563	563 (0)	558 (5)	556 (7)	535 (28)	541 (22)	nd	nd

^a^ Blue-shift values with respect to the λ_max_ in NaP are shown in parenthesis. ^b^ nd = not determined.

**Table 2:** Relative quantum yield of the labeled peptides.

Peptide	QY ^a^ (Variation Relative to NaP pH 7.4)		
	NaP pH 7.4	IPA 50% ^b^	NaAc ^c^ pH 5.0
PyMPO-GKY20	0.080	0.373 (4.66)	nd^d^
mLuc-GKY20	0.008	0.021 (2.63)	nd
Luc-GKY20	0.111	0.639 (5.76)	0.269 (2.42)
aLuc-GKY20	0.202	0.969 (4.80)	nd
Luc-ApoB_L_	0.365	0.389 (1.07)	0.450 (1.23)
aLuc-ApoB_L_	0.574	0.757 (1.32)	nd
Luc-p53pAnt	0.261	0.184 (0.70)	0.418 (1.60)
aLuc-p53pAnt	0.432	0.274 (0.63)	nd
Luc-RGD	0.519	0.443 (0.85)	0.510 (0.98)
aLuc-RGD	0.937	1.040 (1.11)	nd

^a^ Errors ≤ 5% of the reported values. ^b^ Isopropanol:NaP pH 7.4 (1:1, *v*/*v*). ^c^ Sodium acetate 20 mM. ^d^ nd = not determined.

**Table 3:** pK_a_ values of the phenolic group in Luc and Luc-labeled peptides.

Peptide	Net Charge of the Peptidyl Moiety ^a^	pK_a_
Free Luc	not applicable	8.70 ^b^
Luc-RGD	−1	8.40 ± 0.03
Luc-GKY20	+4	8.13 ± 0.04
Luc-ApoB_L_	+6	8.11 ± 0.04
Luc-p53pAnt	+11	7.97 ± 0.03

^a^ Theoretical net charge at pH 7.5–9.0 calculated attributing a charge = +1 to each lysine/arginine residue and a charge = −1 to each aspartate/glutamate residue and to the free C-terminus. ^b^ From reference [6].

## Data Availability

The data presented in this study are available in the article or Supplementary Materials. The raw datasets are available from the corresponding authors upon reasonable request.

## Supplementary Materials

The following are available online at https://www.mdpi.com/article/10.3390/ijms222413312/s1.
