# Supplementary material for: Environment-Sensitive Fluorescent Labelling of Peptides by Luciferin Analogues
Source: Int J Mol Sci. 2021 Dec 10;22(24):13312. doi: 10.3390/ijms222413312 (PMC8706149; doi:10.3390/ijms222413312)
Supplement: Supplementary file 1 [file ijms-22-13312-s001.zip › ijms-1484482 supplementary CORRECT_EN.pdf]

Environment-Sensitive Fluorescent Labelling of Peptides by Luciferin Analogues

Marialuisa Siepi [1,†], Rosario Oliva [2,†], Antonio Masino [1,†], Rosa Gaglione [2], Angela Arciello [2], Rosita Russo [3], Antimo Di Maro [3], Anna Zanfardino [1], Mario Varcamonti [1], Luigi Petraccone [2], Pompea Del Vecchio [2], Marcello Merola [1], Elio Pizzo [1], Eugenio Notomista [1,‡,*] and Valeria Cafaro [1,‡]

[1] Department of Biology, University of Naples Federico II, 80126 Naples, Italy; marialuisa.siepi@unina.it (M.S.); antonio.masino@unina.it (A.M.); anna.zanfardino@unina.it (A.Z.); varcamon@unina.it (M.V.); m.merola@unina.it (M.M.); elipizzo@unina.it (E.P.); vcafaro@unina.it (V.C.)

[2] Department of Chemical Sciences, University of Naples Federico II, 80126 Naples, Italy; rosario.oliva2@unina.it (R.O.); rosa.gaglione@unina.it (R.G.); anarciel@unina.it (A.A.); luigi.petraccone@unina.it (L.P.); pompea.delvecchio@unina.it (P.D.V.)

[3] Department of Environmental, Biological and Pharmaceutical Sciences and Technologies, University of Campania "Luigi Vanvitelli", I-81100 Caserta, Italy; rosita.russo@unicampania.it (R.R.); antimo.dimaro@unicampania.it (A.D.M.)

* Correspondence: notomist@unina.it

† Equally contributing authors.

‡ Also these authors contributed equally.

Content:

Supplementary Methods 2

- **S1. General Methods and Materials** 2
- **S2. Production of recombinant fusion proteins and peptides** 2
- **S3. RP-HPLC analyses** 3
- **S4. Mass spectrometry** 4
- **S5. Labeling of the peptides** 4
- **S6. Antimicrobial activity assay** 5
- **S7. Cytotoxicity assays** 6
- **S8. Liposomes preparation** 6
- **S9. Determination of K_d values and stoichiometries** 7
- **References** 9

Supplementary Tables 10

Supplementary Figures 15

Supplementary Methods

S1. General Methods and Materials

E. coli BL21(DE3) was purchased from Novagen (San Diego, CA, USA). Ni Sepharose™ 6 Fast Flow was from GE Healthcare (Uppsala, Sweden). The (C)RGD peptide was synthesized by CASLO ApS (Lyngby, Denmark) by standard solid-state synthesis. The lipids 1-palmitoyl-2-oleoyl-*sn*-glycero-3-phosphocholine (POPC) and 1-palmitoyl-2-oleoyl-*sn*-glycero-3-*rac*-phosphoglycerol (POPG) were purchased from Avanti Polar Lipids Inc. (Alabaster, AL, USA) and used without any further purification. All other chemicals were from Merck KGaA, Darmstadt, Germany. Concentrations of purified proteins and peptides were determined by spectrophotometric analyses using the extinction coefficients calculated by the ProtParam tool [1] (accessible at the address http://web.expasy.org/protparam/). Extinction coefficients are listed in Table S4. Concentration values of all peptides were also determined by the BCA Protein Assay Kit (Thermo Fisher Scientific, Pierce, Rockford, IL, USA). Sodium Dodecyl Sulphate PolyAcrylamide Gel Electrophoresis (SDS-PAGE) was carried out according to Laemmli [2]. The gel was photographed (ex = 365 nm) immediately after the electrophoresis in the running buffer containing SDS, after repeated washes in isopropanol/water (40% v:v) to remove SDS and after repeated washes in water to remove isopropanol.

S2. Production of recombinant fusion proteins and peptides

Recombinant peptides (C)GKY20, (C)ApoB$_L$ and (C)p53pAnt were prepared by chemical hydrolysis of reduced fusion proteins ONC-DCless-H6-(C)GKY20, ONC-DCless-H6-(C)-ApoB$_L$ and H7-ONC-DCless-(C)p53pAnt, respectively, expressed and purified as previously reported [3]. Briefly, recombinant proteins were expressed in *E. coli* BL21DE3 strain (LB medium, Condalab, Madrid, Spain) as inclusion bodies, purified by immobilized metal affinity chromatography (IMAC) and then dialyzed toward 0.1 M acetic acid. Peptides were released by chemical hydrolysis of the Asp-Cys linker carried out at 60°C for 24 h at pH 2 (18 mM HCl) in the presence of 1 mM Tris(2-carboxyethyl)phosphine hydrochloride (TCEP-HCl).

(C)GKY20 was purified by RP-HPLC with a Jasco LC-4000 system (Tokyo, Japan) equipped with PU-4086 semipreparative pumps and an MD-4010 photo diode array detector, on Europa Protein

300 C18 column (5 µm, 25 × 1) from Teknokroma (Barcelona, Spain) using linear gradient 1 (Supplementary Methods, section S3). Purified peptide was lyophilized and dissolved in 5 mM sodium acetate buffer, pH 5.0. Sample was stored at −80 °C under nitrogen atmosphere. Purity of peptide was evaluated by RP-HPLC on Europa Protein 300 C18 column. Peptide was eluted by linear gradients 2 (Supplementary Methods, section S3).

(C)ApoB$_L$ and (C)p53pAnt were purified by column-free procedure based on the different solubility of the carrier and peptides at pH 7 [3]. To this purpose, hydrolysis mixtures were neutralized by adding diluted NH_3 in the presence of 2 mM TCEP for 1 h at 28 °C under nitrogen atmosphere. Uncleaved fusion proteins and the carrier, insoluble at pH 7, were separated from soluble peptides by centrifugation (1 h, 18,000g at 4 °C). Supernatants, containing soluble peptides, were immediately incubated with the appropriate thiol-modifying reagent to label N-terminal cysteine residues as described below. Purity of peptides was evaluated by RP-HPLC on Europa Protein 300 C18 column by gradient 1 (Supplementary Methods, section S3). The identity of the peptides was confirmed by mass spectrometry analyses (Supplementary Methods, section S4; Table S1). Recombinant peptides (P)GKY20 and (P)ApoB$_L$ were prepared as previously described [4,5].

S3. RP-HPLC analyses

0.05% trifluoracetic acid (TFA) in water (solvent A) and 0.05% TFA in acetonitrile (solvent B) were used as solvents. Elution profiles were recorded by three different linear gradients as follows:

gradient 1: from 5% to 25% solvent B in 10 min, from 25% to 35% solvent B in 30 min, from 35% to 50% solvent B in 10 min, from 50% to 100% solvent B in 10 min, isocratic elution at 100% solvent B for 10 min. The elution was monitored at 280 and 220 nm at a flow rate of 2 ml/min;

gradient 2: isocratic elution at 5% solvent B for 10 min, from 5% to 20% solvent B in 5 min, from 20% to 40% solvent B in 30 min, from 40% to 50% solvent B in 10 min, from 50% to 100% solvent B in 5 min, isocratic elution at 100% solvent B for 7 min. The elution was monitored at 280 nm at a flow rate of 2 ml/min;

gradient 3: isocratic elution at 5% solvent B for 10 min, from 5% to 20% solvent B in 5 min, from 20% to 40% solvent B in 30 min, from 40% to 50% solvent B in 10 min, from 50% to 95% solvent B in 1 min. The elution was monitored at 280 and 408 nm at a flow rate of 1 ml/min;

gradient 4: isocratic elution at 5% solvent B for 10 min, from 5% to 20% solvent B in 5 min, from 20% to 40% solvent B in 60 min, from 40% to 50% solvent B in 10 min, from 50% to 95% solvent B in 1 min, isocratic elution at 95% solvent B for 14 min . The elution was monitored at 280 and 330 nm at a flow rate of 1 ml/min;
gradient 5: from 0% to 30% solvent B in 60 min, from 30% to 50% solvent B in 10 min, from 50% to 100% solvent B in 1 min, isocratic elution at 100% solvent B for 10 min. The elution was monitored at 280 nm at a flow rate of 2 ml/min.

S4. Mass spectrometry

MALDI-TOF micro MX spectrometer (Waters, Manchester, UK) was used to verify protein and peptide identity. 1 μL of each sample was mixed with 1 μL of saturated α-cyano-4-hydroxycinnamic acid matrix solution [10 mg/mL in acetonitrile:0.1% TFA (1:1; v/v)]. Thus, a droplet of the resulting mixture (1 μL) was placed on the mass spectrometer's sample target and dried at room temperature. Once the liquid was completely evaporated, samples were loaded into the mass spectrometer and analysed. The instrument was externally calibrated using a tryptic alcohol dehydrogenase digest (Waters, Manchester, UK) in reflectron mode. For linear mode, a four-point external calibration was applied using an appropriate mixture (10 pmol/mL) of insulin, cytochrome C, horse Mb and trypsinogen as standard proteins (Merck KGaA, Darmstadt, Germany). A mass accuracy near to the nominal (50 and 300 ppm in reflectron and linear modes, respectively), was achieved for each standard. All spectra were processed and analysed using MassLynx 4.0 software.

S5. Labeling of the peptides

Purified (C)GKY20 (0.5 mg, 100 μM final concentration) was labeled by the thiol reactive fluorescent dye PyMPO maleimide (1-[2-(maleimido)ethyl]-4-[5-(4-methoxyphenyl)-2-oxazolyl]pyridinium triflate) and by three 2-cyanobenzothiazoles, 6-hydroxy-2-cyanobenzothiazole (CBT-OH), 6-amino-2-cyanobenzothiazole (CBT-NH_2) and 6-methoxy-2-cyanobenzothiazole (CBT-OCH_3). Labeling was performed following the procedure previously described [3], with minor modifications. Briefly, PyMPO labeling was carried out in 1 mL of 0.1 M Tris-HCl buffer, pH 7.2, containing 7 mM EDTA, 2 M guanidine-HCl, 1 mM TCEP and 1 mM PyMPO maleimide. Labeling with CBTs was performed

in 15 mM sodium phosphate buffer (NaP), pH 7.4, containing 2 M guanidine-HCl, 1 mM TCEP and 1 mM CBT (10x stock solutions in dimethylformamide). The molar ratio TCEP: CBT (or PyMPO): free thiols was 10:10:1. Samples were incubated at 25°C for 2 h, in the dark under nitrogen atmosphere. Control mixtures without peptide were also prepared.

Reaction yields were monitored by RP-HPLC on C18 columns. PyMPO and CBT-OCH_3 Labeling reactions were monitored on Jupiter 5u C18 300A column (250 × 4,6 mm, 5 μm particle size, provided by Phenomenex, Torrance, CA, USA), using linear gradient 3 (Supplementary Methods, section S3). CBT-OH and CBT-NH_2 labeling reactions were monitored on Europa Protein 300 C18 column using linear gradient 2 (Supplementary Methods, section S3). To purify labeled peptides, 1% TFA was added to reaction mixtures and the samples were loaded onto C18 columns equilibrated in water/0.05% TFA (solvent A). All labeled peptides were purified using the same column and gradient used to monitor the labeling reactions, except mLuc-GKY20 which was purified using linear gradient 4 (Supplementary Methods, section S3).

Synthetic peptide (C)RGD (1 mg, about 0.5 mM final concentration) was labeled by using CBT-OH and CBT-NH_2 following the same procedure described above. The molar ratio of TCEP and CBT over thiols was 5:5:1. Reactions were monitored by RP-HPLC on Europa Protein 300 C18 column using gradient 5 (Supplementary Methods section S3). Labeled peptides (Luc-RGD and aLuc-RGD) were purified on the same column using gradient 5 (Supplementary Methods, section S3).

Recombinant peptides (C)ApoB$_L$ and (C)p53pAnt were labeled following a procedure similar to that above described for (C)GKY20 peptide, with minor modifications. Recombinant peptides were purified by selective precipitation of carrier and fusion proteins as above described. Supernatants, containing purified peptides (4 mg, 50 μM final concentration), were incubated with CBT-OH and CBT-NH_2 (0.5 mM) in 15 mM NaP buffer, pH 7.4, containing 2 M guanidine-HCl, 2 mM TCEP, for 2 h at 25°C, in the dark under nitrogen atmosphere. (C)ApoB$_L$ and (C)p53pAnt reactions were monitored by RP-HPLC on Europa Protein 300 C18 column using gradient 1 and 5, respectively (Supplementary Methods, section S3). To simplify the peptide HPLC purifications, Luc-ApoB$_L$, aLuc-ApoB$_L$ and aLuc-p53pAnt reactions were quenched by L-cysteine with molar excess of 5:1 on CBT, for 1 h at 37 °C in the dark. Peptides were purified on Europa Protein 300 C18 column using gradient 1 (Luc-

ApoB$_L$ and aLuc-ApoB$_L$) and gradient 5 (Luc-p53pAnt and aLuc-p53pAnt) (Supplementary Methods, section S3).

All the labeled peptide after purification were lyophilized and dissolved in water. Purity was verified by RP-HPLC on C18 column. Peptide identity was assessed by mass spectrometry analyses (Supplementary Methods, section S4; Table S1).

S6. Antimicrobial activity assay

MIC (Minimum Inhibitory Concentration) analyses were performed by broth microdilution method for antimicrobial peptides previously described [4] with minor modifications. In details, assays were carried out in Nutrient Broth 0.5x (Difco, Detroit, MI, USA) using sterile 96-well polypropylene microtiter plates (cat. 3879, Costar Corp., Cambridge, MA, USA). Bacterial strains were grown in Luria-Bertani (LB) medium overnight at 37°C and then diluted in Nutrient Broth at a final concentration of ~$5x10^5$ CFU/mL per well. Twofold serial dilutions of peptides were carried out in the test wells to obtain concentrations ranging from 50 μM to 0.05 μM (Luc- and aLuc-GKY20) and from 40 μM to 0.04 μM (Luc- and aLuc-ApoB$_L$). Plates were incubated overnight at 37°C. MIC value was taken as the lowest concentration at which growth was inhibited. Three independent experiments were performed for each MIC value. The peptide antibiotic polymyxin B and vancomycin (Merck KGaA, Darmstadt, Germany) were tested as control (twofold serial dilutions starting from 64 μg/mL concentration). MIC values were measured on Gram-negative (*Escherichia coli* ATCC 25922) and Gram-positive strains (*Staphylococcus aureus* ATCC 6538P and *Bacillus globigii* TNO BMO13).

S7. Cytotoxicity assays

Human cervical cancer cells (HeLa) and human noncancerous immortalized keratinocyte cells (HaCaT) were purchased from American Type Culture Collection (ATCC) and were cultured in Dulbecco's modified Eagle's medium (Euroclone, Milan, Italy), supplemented with 10% fetal bovine serum (HyClone Laboratories Inc., South Logan, UT, USA), 2 mM L-glutamine and antibiotics. HeLa and HaCaT cells were grown in a 5% CO_2 humidified atmosphere at 37°C. Cells were seeded in 96-well plates at a density of 2.5×10^3/well. 24 h after seeding, cells were incubated with increasing amount of the peptides under test (from 10 to 30 μM) for 48 h. At the end of incubation, cell viability

was assessed by the MTT assay as previously described [5]. Cell survival was expressed as percentage of viable cells in the presence of the analysed peptides, with respect to control cells. Control cells are represented by cells grown in the absence of peptides and by cells supplemented with identical volumes of protein buffer (10 mM sodium phosphate buffer, pH 7.4). Each sample was tested in three independent analyses, each carried out in triplicate. Quantitative parameters were expressed as the mean value ± SD. Significance was determined by Student's t-test at a significance level of 0.05.

S8. Liposomes preparation

An appropriate amount of lipids (POPC and POPG) was accurately weighted in a glass vial and dissolved in a chloroform/methanol mixture (2:1 v:v). The solution was gently vortexed to ensure their complete dissolution in the organic solvent. Then, the organic solvent was removed by gentle evaporation with nitrogen gas in order to obtain a thin lipid film. To remove final traces of organic solvent, the sample was placed under vacuum overnight. Then, the sample was hydrated with an appropriate volume of 10 mM phosphate buffer, pH 7.4, and vigorously mixed, obtaining a suspension of multilamellar vesicles (MLVs). In order to produce large unilamellar vesicles (LUVs), the extrusion method was applied. Briefly, LUVs were produced using a Mini-Extruder (Avanti Polar Lipid Inc., Merck KGaA, Darmstadt, Germany) passing the MLV suspension through a 100 nm pore size polycarbonate membrane twenty-one times. Dynamic light scattering (DLS) measurements were performed to check the size of the vesicles after extrusion. DLS experiments were performed by means of a Zetasizer nano-ZS (Malvern Instruments, Malvern, UK). The average hydrodynamic radius was found to be around 120 nm, which is consistent with the formation of unilamellar vesicles. Vesicles composed of POPC and POPC/POPG (4:1 molar ratio) were prepared.

S9. Determination of K_d values and stoichiometries

To study the interaction of peptides with LPS we started from the simple model:

$$P + S \leftrightarrows PS$$

where P is the fluorescent peptide and S is a generic binding site on LPS micelles (or another type of receptor). We assumed that all the binding sites were equivalent and independent. The K_d will be given by:

$$K_D = [P][S] / [PS] \quad (1)$$

Indicating with PT and ST the total concentrations of P and S, respectively:

$$P_T = [P] + [PS]$$

$$S_T = [S] + [PS]$$

Equation 1 can be rewritten as:

$$[PS]^2 - (S_T + P_T + K_D)\,[PS] + P_T\,S_T = 0 \quad (2)$$

From equation 2 it is possible to obtain [PS]:

$$[PS] = [(S_T + P_T + K_D) - SQR((S_T + P_T + K_D)^2 - 4\,P_T\,S_T)]/2 \quad (3)$$

Moreover, the total fluorescence of the sample will be the sum of the fluorescence of free, $F_{(P)}$, and bound peptide, $F_{(PS)}$:

$$F = F_{(P)} + F_{(PS)} \quad (4)$$

Defining:

MF_{free} = molar fluorescence of free peptide, P,

and

MF_{bnd} = molar fluorescence of bound peptide, PS,

equation 4 can be rewritten as:

$$F = MF_{free}\,[P] + MF_{bnd}\,[PS] \quad (5)$$

Or as:

$$F = MF_{free}\,(P_T - [PS]) + MF_{bnd}\,[PS] \quad (6)$$

Equation 6 can be rearranged to give:

$$F = MF_{free}\,P_T + (MF_{bnd} - MF_{free})\,[PS] \quad (7)$$

From equations 3 and 7:

$$F = MF_{free}\,P_T + (MF_{bnd} - MF_{free})\,[(S_T + P_T + K_D) - SQR((S_T + P_T + K_D)^2 - 4\,P_T\,S_T)]/2 \tag{8}$$

Equation 8 provides the expected fluorescence either as function of S_T at a constant concentration of peptide (P_T = constant), or as function of P_T at a constant concentration of the receptor (S_T = constant). Working at constant LPS concentration (S_T = constant) and variable concentration of the labelled peptide, equation 8 describes a curve tangent to the straight line "$F = MF_{bnd}\,P_T$" (fluorescence expected if all the peptide is in the bound state), and with an asymptote, defined by:

$$F = (MF_{bnd} - MF_{free})\,S_T + MF_{free}\,P_T \tag{9}$$

which is parallel to the straight line "$F = MF_{free}\,P_T$" (fluorescence expected if all the peptide is in the free state).

The asymptote intersects the x axis at:

$$(MF_{free} - MF_{bnd})\,S_T / MF_{free}$$

When $MF_{bnd} = 0$ (or << MF_{free}) the curve is tangent to the x axis and the asymptote intersects the x axis at S_T as shown in the scheme below. The K_d value determines how close the curve is to the interception between the asymptote and the x axis. K_d and stoichiometry values reported in Table 4 and the curves in Fig. 7 of the main text were obtained implementing equation 8 in Graphpad Prism, setting MF_{bnd} = costant = 0.

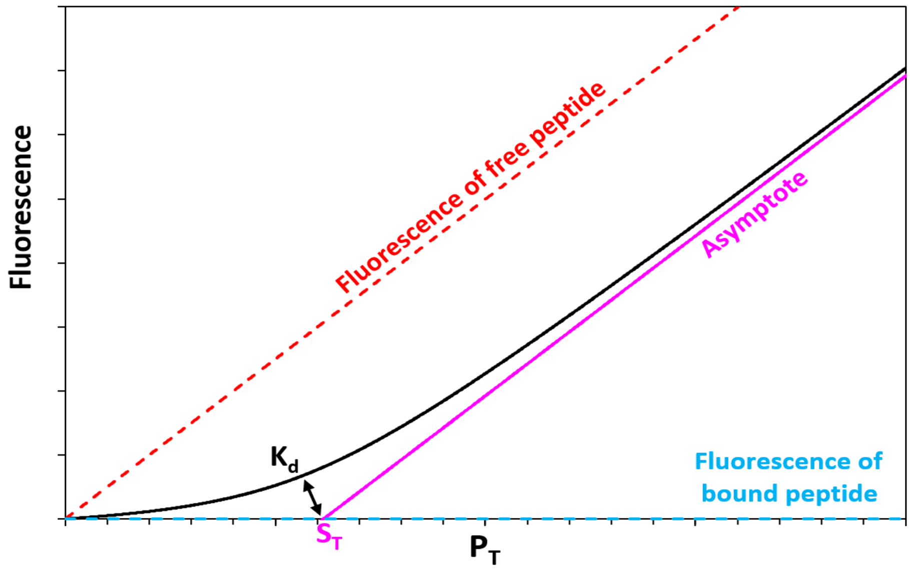

References

1. E. Gasteiger, C. Hoogland, A. Gattiker, S. Duvaud, M.R. Wilkins, R.D. Appel, A. Bairoch, Protein Identification and Analysis Tools on the ExPASy Server BT - The Proteomics Protocols Handbook, in: J.M. Walker (Ed.), Humana Press, Totowa, NJ, 2005: pp. 571–607. doi:10.1385/1-59259-890-0:571.
2. U.K. Laemmli, Cleavage of Structural Proteins during the Assembly of the Head of Bacteriophage T4, Nature. 227 (1970) 680–685. doi:10.1038/227680a0.
3. K. Pane, M. Verrillo, A. Avitabile, E. Pizzo, M. Varcamonti, A. Zanfardino, A. Di Maro, C. Rega, A. Amoresano, V. Izzo, A. Di Donato, V. Cafaro, E. Notomista, Chemical Cleavage of an Asp-Cys Sequence Allows Efficient Production of Recombinant Peptides with an N-Terminal Cysteine Residue, Bioconjug. Chem. 29 (2018) 1373–1383. doi:10.1021/acs.bioconjchem.8b00083.
4. K. Pane, L. Durante, E. Pizzo, M. Varcamonti, A. Zanfardino, V. Sgambati, A. Di Maro, A. Carpentieri, V. Izzo, A. Di Donato, V. Cafaro, E. Notomista, Rational design of a carrier protein for the production of recombinant toxic peptides in Escherichia coli, PLoS One. 11 (2016) e0146552. doi:10.1371/journal.pone.0146552.
5. R. Gaglione, E. Dell'Olmo, A. Bosso, M. Chino, K. Pane, F. Ascione, F. Itri, S. Caserta, A. Amoresano, A. Lombardi, H.P. Haagsman, R. Piccoli, E. Pizzo, E.J.A. Veldhuizen, E. Notomista, A. Arciello, Novel human bioactive peptides identified in Apolipoprotein B: Evaluation of their therapeutic potential, Biochem. Pharmacol. 130 (2017) 34–50. doi:10.1016/j.bcp.2017.01.009.

Supplementary Tables

Table 1. Theoretical and experimental molecular weights of proteins and peptides.

	Theoretical MW (Da)	Experimental MW (Da)	Length[a]
**FUSION PROTEIN**			
ONC-DCless-H6-(C)GKY20[b]	15949.33	15951.64	135
ONC-DCless-H6-(C)ApoB$_L$[b]	17425.16	17425.24	152
H7-ONC-DCless-(C)p53pAnt[b]	17961.54	17958.87	152
**PEPTIDE**			
(C)GKY20	2615.18	2614.16	21
(C)ApoB$_L$	4080.98	4083.17	38
(C)p53pAnt	4537.26	4536.56	38
PyMPO-(C)GKY20	2991.42	2991.30	21
(C)RGD	690.74	690.53[c]	7
Luc-GKY20	2774.37	2774.3	21
aLuc-GKY20	2773.39	2773.24	21
mLuc-GKY20	2788.37	2788.35	21
Luc-ApoB$_L$	4240.17	4235.67	38
aLuc-ApoB$_L$	4239.19	4238.59	38
Luc-p53pAnt	4696.45	4695.02	38
aLuc-p53pAnt	4695.47	4695.14	38
Luc-RGD	849.92	850.22	7
aLuc-RGD	848.94	849.29	7

[a]Number of amino acids.
[b]Protein in the reduced form.
[c]Molecular weight determined by the provider (CASLO ApS, Lyngby, Denmark).

Table S2. Molecular properties of some common fluorescent labels for peptides.

Fluorescent label[a]	Conjugation mode[b]	MW (Da)	ASA[c] (Å²)	P-ASA[d] (Å²)	P-ASA/ ASA	+/-[e]	net charge[f]	Fluorophore type/name
	**N-terminus**							
**NBD**	**Φ-NH-P**	**165.1**	**299.9**	**128.6**	0.43	**0/0**	**0**	**4-nitrobenzofurazano**
Pacific Blue	Φ-C(O)-NH-P	226.1	331.1	222.1	0.67	0/1	-1	difluorohydroxycoumarin
**NBD-Glycine**	**Φ-C(O)-NH-P**	**222.2**	**384.5**	**181.8**	**0.47**	**0/0**	**0**	**4-nitrobenzofurazano**
(7-methoxycoumarin-4-yl)acetyl	Φ-C(O)-NH-P	218.2	395.0	105.3	0.27	0/0	0	methoxycoumarin
**Luciferin**	**Φ-C(O)-NH-P**	**264.3**	**408.4**	**138.2**	**0.34**	**0/1**	**~-0.1[g]**	**luciferin**
**amino-Luciferin**	**Φ-C(O)-NH-P**	**263.3**	**412.0**	**126.9**	**0.31**	**0/0**	**0**	**luciferin**
coumarin 343	Φ-C(O)-NH-P	269.3	414.5	84.4	0.20	0/0	0	aminocoumarin
**Dansyl Chloride**	**Φ-S(O₂)-NH-P**	**235.3**	**420.7**	**135.4**	**0.32**	**0/0**	**0**	**dansyl**
Alexa Fluor™ 350	Φ-C(O)-NH-P	296.3	432.8	221.6	0.51	0/1	-1	aminocoumarin
**Pyrene-1-butirrate**	**Φ-C(O)-NH-P**	**272.3**	**463.8**	**42.2**	**0.09**	**0/0**	**0**	**pyrene**
Fluorescein-(5)-carboxylate	Φ-C(O)-NH-P	358.3	474.1	173.1	0.37	0/2	-2	Fluorescein
**NBD-hexanoate**	**Φ-C(O)-NH-P**	**278.2**	**494.7**	**177.7**	**0.36**	**0/0**	**0**	**4-nitrobenzofurazano**
BODIPY FL	Φ-C(O)-NH-P	276.0	502.0	77.5	0.15	0/0	0	BODIPY
BODIPY TR	Φ-C(O)-NH-P	408.2	550.2	90.3	0.16	0/0	0	BODIPY
**Dapoxyl Carboxylic Acid**	**Φ-C(O)-NH-P**	**292.3**	**557.8**	**71.4**	**0.13**	**0/0**	**0**	**Dapoxyl**
Atto 495	Φ-C(O)-NH-P	336.4	572.6	39.7	0.07	**1/0**	**1**	dimethylaminoacrine
BODIPY TMR	Φ-C(O)-NH-P	382.2	617.0	91.7	0.15	0/0	0	BODIPY
TAMRA-(5)-carboxylate	Φ-C(O)-NH-P	414.4	636.3	94.5	0.15	1/1	0	rhodamine
Alexa Fluor™ 430	Φ-C(O)-NH-P	486.5	654.9	263.1	0.40	0/1	-1	aminocoumarin
**PyMPO, SE**	**Φ-C(O)-NH-P**	**371.4**	**662.4**	**104.4**	**0.16**	**1/0**	**1**	1-(3-(succinimidyloxycarbonyl)benzyl)-4-(5-(4-methoxyphenyl)oxazol-2-yl)pyridinium bromide
Rhodamine B	Φ-C(O)-NH-P	427.5	696.8	30.3	0.04	1/0	1	rhodamine

**cascade yellow**	**Φ-C(O)-NH-P**	**450.5**	**732.4**	**258.1**	**0.35**	**1/1**	**0**	**5-{2-[1-(3-{[(2,5-dioxopyrrolidin-1-yl)oxy]carbonyl}benzyl)pyridinium-4-yl]-1,3-oxazol-5-yl}-2-methoxybenzene-sulfonate**
Alexa Fluor™ 405	Φ-C(O)-NH-P	608.6	748.5	380.1	0.51	0/3	-3	hydroxypyrene-trisulfonate derivative

Continued on next page

Fluorescent label[a]	Conjugation mode[b]	MW (Da)	ASA[c] (Å^2)	P-ASA[d] (Å^2)	P-ASA/ ASA	+/-[e]	net charge[f]	Fluorophore type/name
	**Cys-side chain**							
**bimane**	**Φ-CH$_2$-S-P**	**192.2**	**381.1**	**62.1**	**0.16**	**0/0**	**0**	**bimane**
**N-(1-pyrene)-maleimide**	**Φ-Suc-S-P**	**299.3**	**440.1**	**42.4**	**0.10**	**0/0**	**0**	**pyrene**
**BADAN**	**Φ-CH$_2$-S-P**	**231.3**	**448.7**	**51.1**	**0.11**	**0/0**	**0**	(6-bromoacetyl-2-dimethylaminonaphthalene)
**IANBD**	**Φ-CH$_2$-S-P**	**293.2**	**465.8**	**143.3**	**0.31**	**0/0**	**0**	NBD analogue: (N,N'-dimethyl-N-(iodoacetyl)-N'-(7-nitrobenz-2-oxa-1,3-diazol-4-yl)ethylenediamine)
**1,5-IAEDANS**	**Φ-CH$_2$-S-P**	**307.3**	**516.8**	**178.6**	**0.35**	**0/1**	**-1**	(5-((((2-iodoacetyl)amino)ethyl)amino)naphthalene-1-sulfonic Acid)
5-(iodoacetamido)fluorescein	Φ-CH$_2$-S-P	387.3	532.4	175.1	0.33	0/2	-2	fluorescein
fluorescein-5-maleimide	Φ-Suc-S-P	427.4	549.8	180.6	0.33	0/2	-2	fluorescein
**4-DMN-maleimide**	**Φ-Suc-S-P**	**365.4**	**569.8**	**101.0**	**0.18**	**0/0**	**0**	4-N,N-dimethylamino-1,8-naphthalimide
**Dansyl-aminoethyl-maleimide**	**Φ-Suc-S-P**	**375.4**	**602.4**	**118.6**	**0.20**	**0/0**	**0**	**dansyl**
**PyMPO maleimide**	**Φ-Suc-S-P**	**378.4**	**661.8**	**110.3**	**0.17**	**1/0**	**1**	**PyMPO**

BODIPY FL-maleimide	Φ-Suc-S-P	416.2	704.9	111.8	0.16	0/0	0	BODIPY
Atto 495-maleimide	Φ-Suc-S-P	476.6	769.1	74.3	0.10	**1/0**	**1**	dimethylaminoacrine
Alexa Fluor 488-C5-maleimide	Φ-Suc-S-P	684.6	838.9	388.3	0.46	1/3	-2	rhodamine

[a] Fluorescent labels with environment dependent behavior are shown in bold. Fluorescent labels are ordered from top to bottom according to their ASA.

[b]Φ-, fluorescent moiety of the label; NH-P, N-terminus of the peptide; S-P, sulfur of a cysteine residue; C(O), S(O2), CH_2, Suc, carbonyl, sulfonyl, methylene and succinimidyl group, respectively, of the label.

[c]Solvent accessible surface area of the label.

[d]Polar-accessible surface area.

[e]Number of positively and negatively charged/ionizable groups in the label.

[f]Approximate net charge at neutral pH.

[g]Estimated assuming a pK_a = ~8.

Table S3. Antimicrobial activity expressed as MIC values.

	Strain		
	Escherichia coli **25922 ATCC**	*Staphylococcus aureus* **ATCC 6538P**	*Bacillus globigii* **TNO BMO13**
**Peptide**	**MIC (μM)[a]**		
(P)GKY20	3.12	1.56	nd[b]
Luc-GKY20	3.12	1.56	nd
aLuc-GKY20	6.25	1.56	nd
(P)ApoB$_L$	2.5	nd	2.5
Luc-ApoB$_L$	2.5	nd	1.25
aLuc-ApoB$_L$	5.0	nd	5.0
**Antibiotic (positive control)**	**MIC (μg/mL)[a]**		
Vancomycin	nd	0.5	2.0
Polymyxin B	0.5	nd	nd

[a]Data were obtained from a minimum of three independent experiments. The highest value was accepted as MIC. A difference of one scalar dilution is generally considered not significant.

[b]Not determined.

Table S4. Extinction coefficients calculated by ProtParam[a].

	ε_{280} (M^{-1} cm^{-1})	$\varepsilon_{280}^{(0.1\%)}$ (mg/mL)
**Proteins**		
ONC-DCless-H6-(C)GKY20	24,410	1.53
ONC-DCless-H6-(C)$ApoB_L$	15,930	0.914
H7-ONC-DCless-(C)p53pAnt	26,930	1.499
**Peptides**		
(C)GKY20	8,480	3.250
(C)p53pAnt	11,000	2.42

[a] https://web.expasy.org/protparam/

GKY20 GKYGFYTHVFRLKKWIQKVI

$ApoB_L$ HVALKPGKLKFIIPSPKRPVKLLSGGNTLHLVSTTKT

p53pAnt GSRAHSSHLKSKKGQSTSRHKKWKMRRNQFWVKVQRG

antennipedia

RGD GRGDSP

Figure S1. Peptide sequences colored by residue type (blue, basic; cyan, histidine; red, acidic; green, hydrophobic; yellow polar; gray borderline).

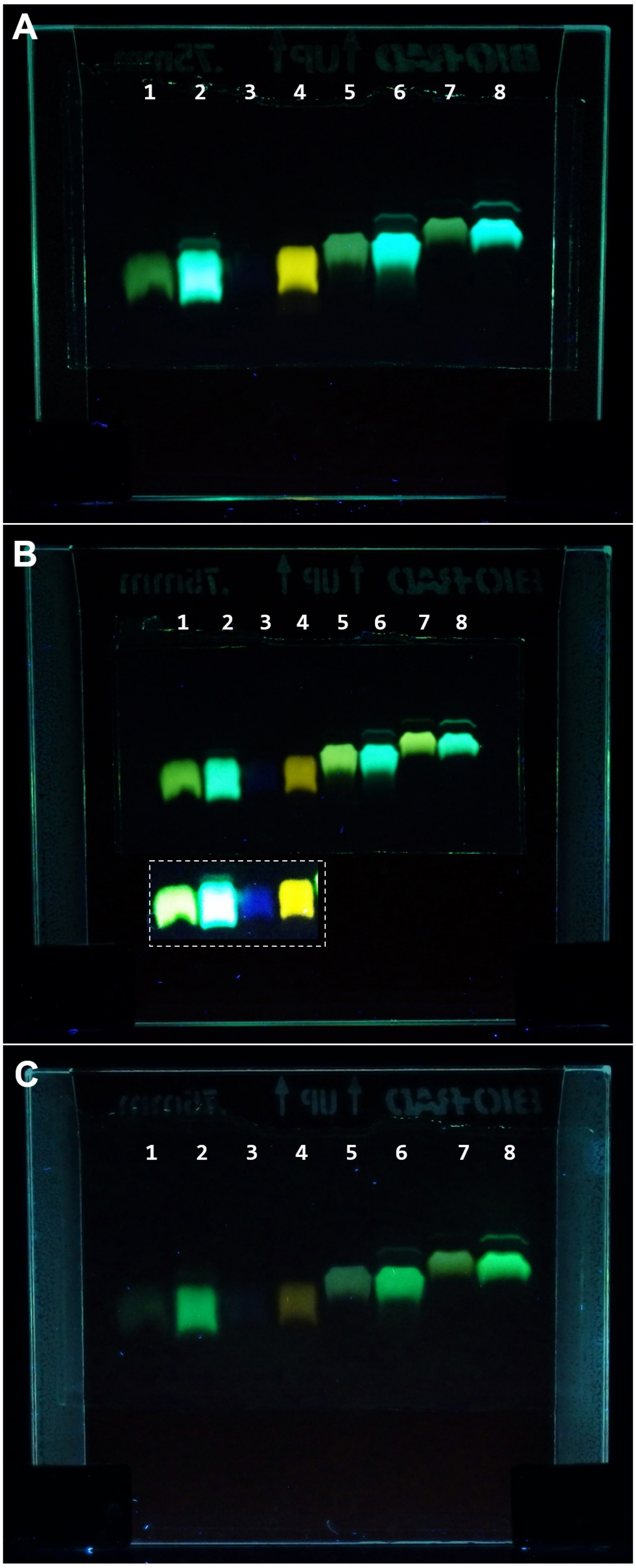

Figure S2. SDS-PAGE analysis of the labeled peptides. Lanes 1-8, Luc-GKY20, aLuc-GKY20, mLuc-GKY20, PyMPO-(C)GKY20, Luc-ApoBL, aLuc-ApoBL, Luc-p53pAnt, aLuc-p53pAnt. The gel was photographed immediately after the analysis in the running buffer containing SDS (**A**), after repeated washes in isopropanol/water (40% v:v) to remove SDS (**B**) and after repeated washes in water to remove isopropanol (**C**). The inset in (B) shows lanes 1-4 with increased contrast to highlight the weak blue fluorescence of mLuc-GKY20.

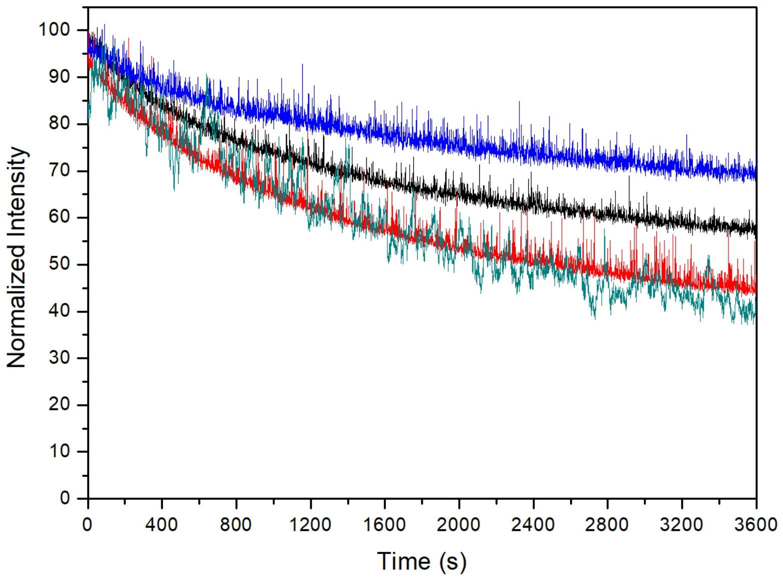

Figure S3. Relative fluorescence emission (%) of labelled GKY20 variants as function of time under constant excitation at the respective λ_{max}. Blue line, Luc-GKY20; red line, aLuc-GKY20; teal line, mLuc-GKY20; black line, PyMPO-(C)GKY20.

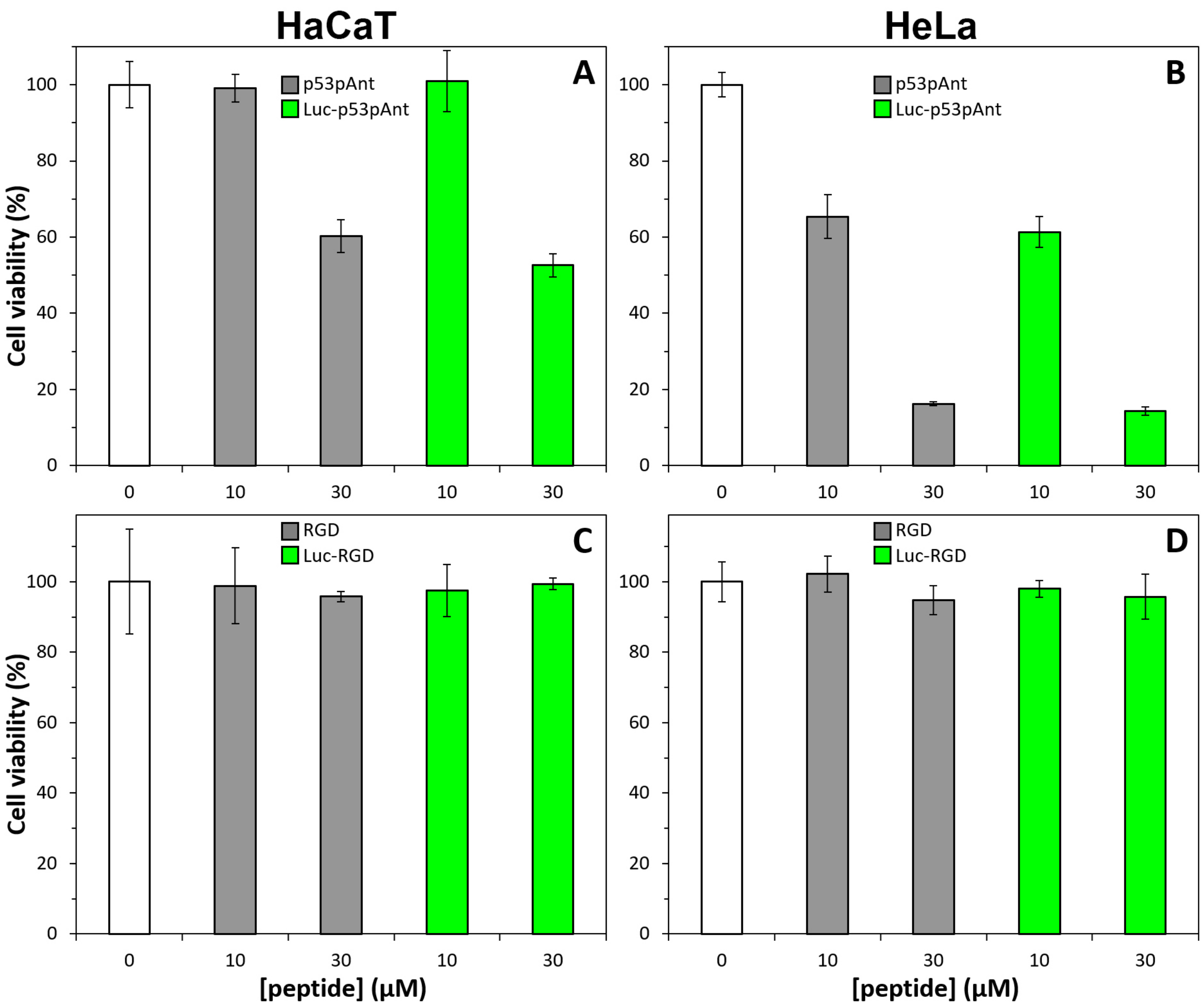

Figure S4. Effects of unlabeled and labeled peptides p53pAnt (**A**, **B**) and RGD (**C**, **D**) on HaCaT (immortalized keratinocytes) and HeLa (cervical cancer cells).

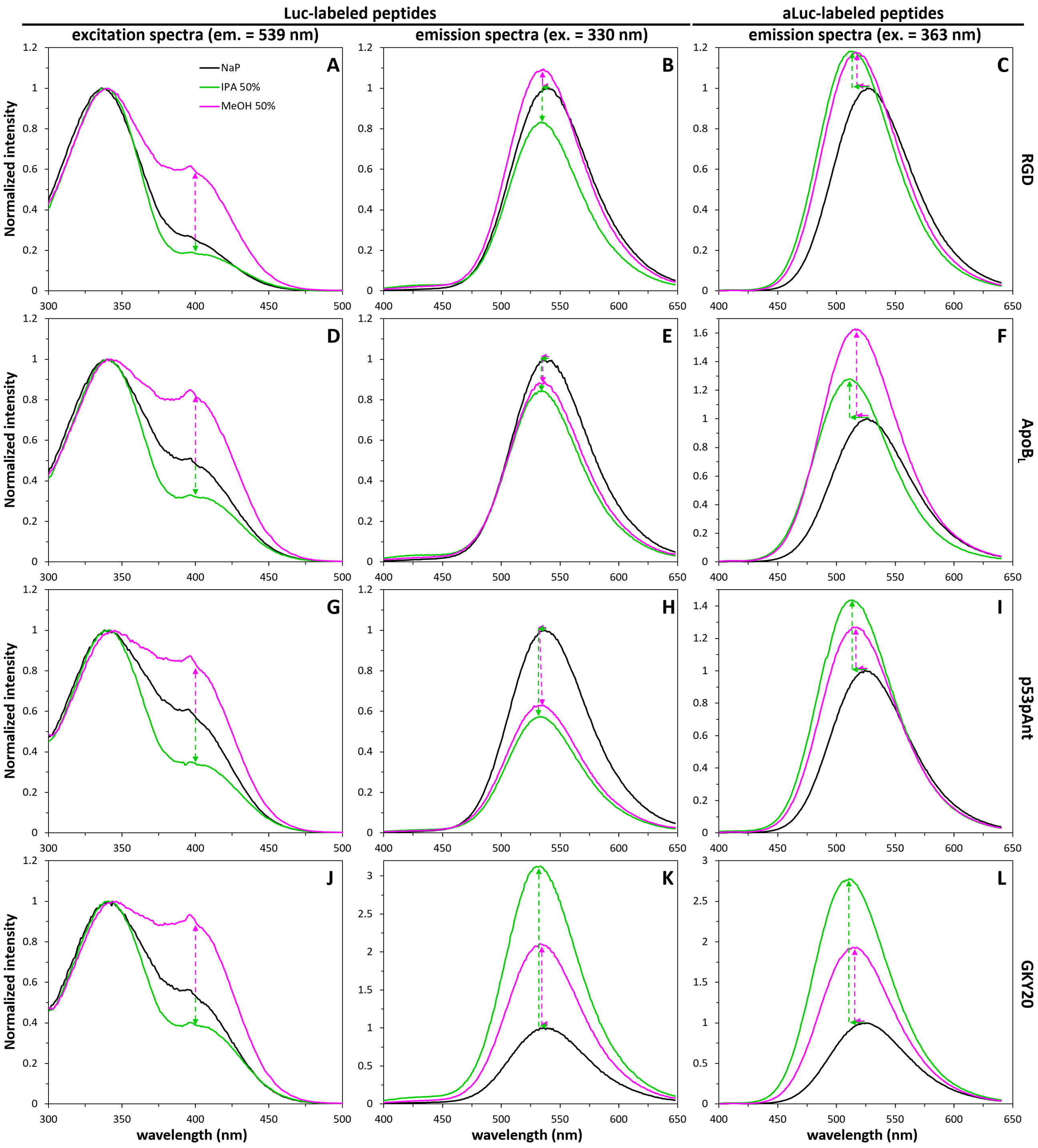

Figure S5. Fluorescence of labeled peptides in 50% methanol or isopropanol in NaP pH 7.5 (v:v). Emission spectra recorded in the presence of organic solvents were normalized to the corresponding spectra in NaP. Arrows highlight the main changes with respect to NaP.

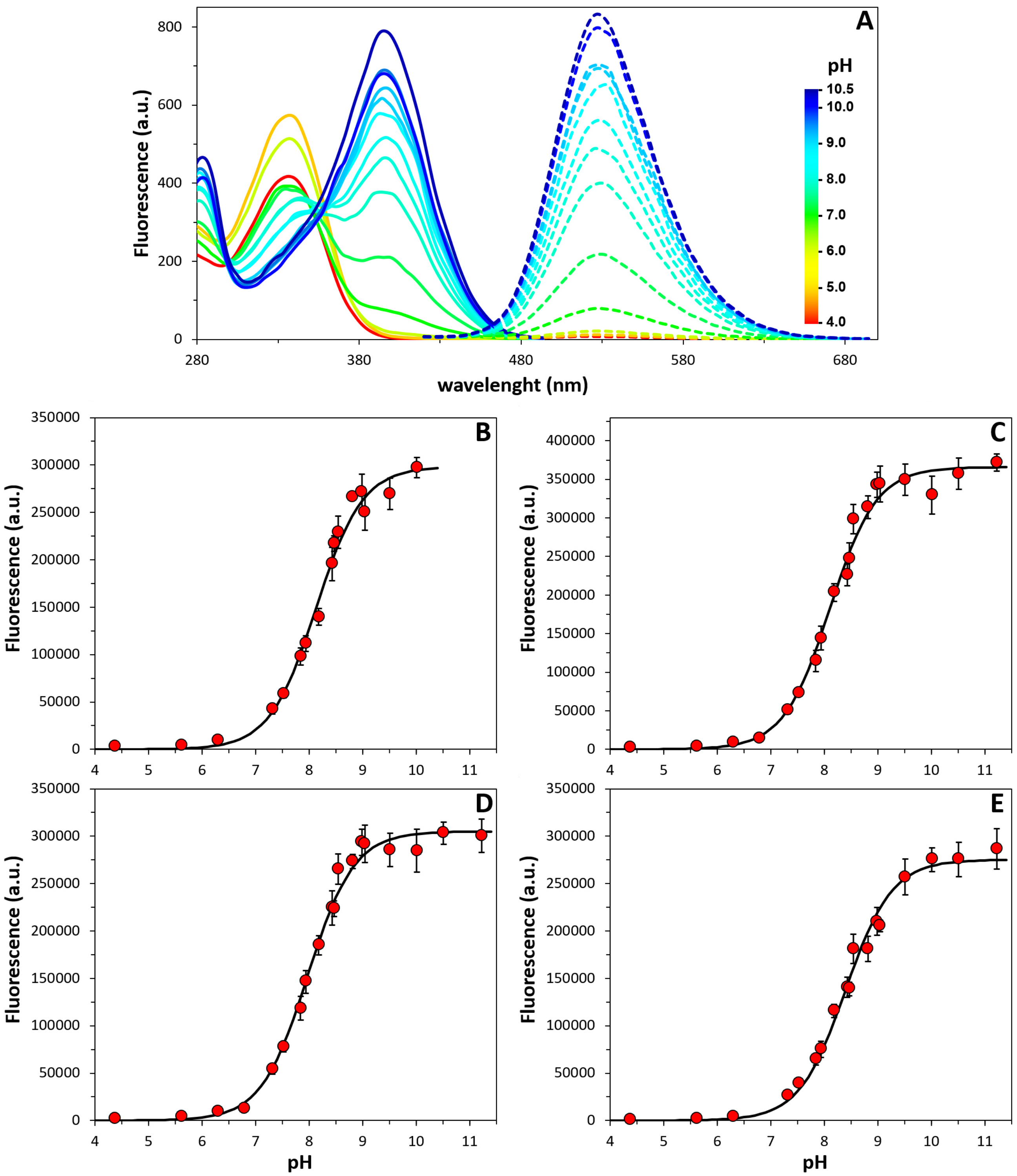

Figure S6. Response of Luc-labeled peptides to pH. (**A**) Excitation (solid lines) and emission (dashed line) spectra of Luc-GKY20 as function of pH. Excitation spectra were recorded at 539 nm; emission spectra were recorded after excitation at 400 nm. (**B**–**E**) Determination of the pK_a values of Luc-GKY20, Luc-ApoB$_L$, Luc-p53pAnt and Luc-RGB, respectively (ex. = 400 nm; em. = 539 nm).

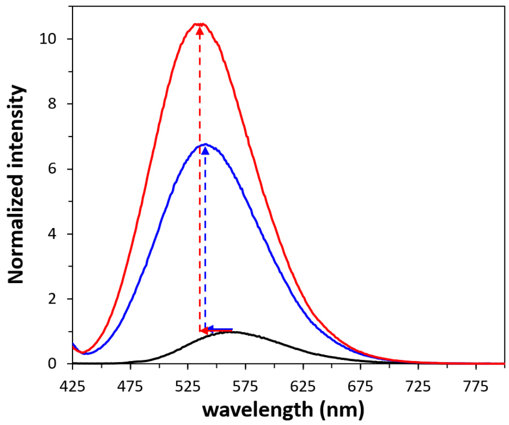

Figure S7. Fluorescence of PyMPO-(C)GKY20 in the presence of liposomes. Black line, NaP pH 7.5; blue line, POPC; red line, POPC/POPG (5:1). Spectra recorded in the presence of liposomes were normalized to the corresponding spectra in NaP. Arrows highlight the main changes with respect to NaP.

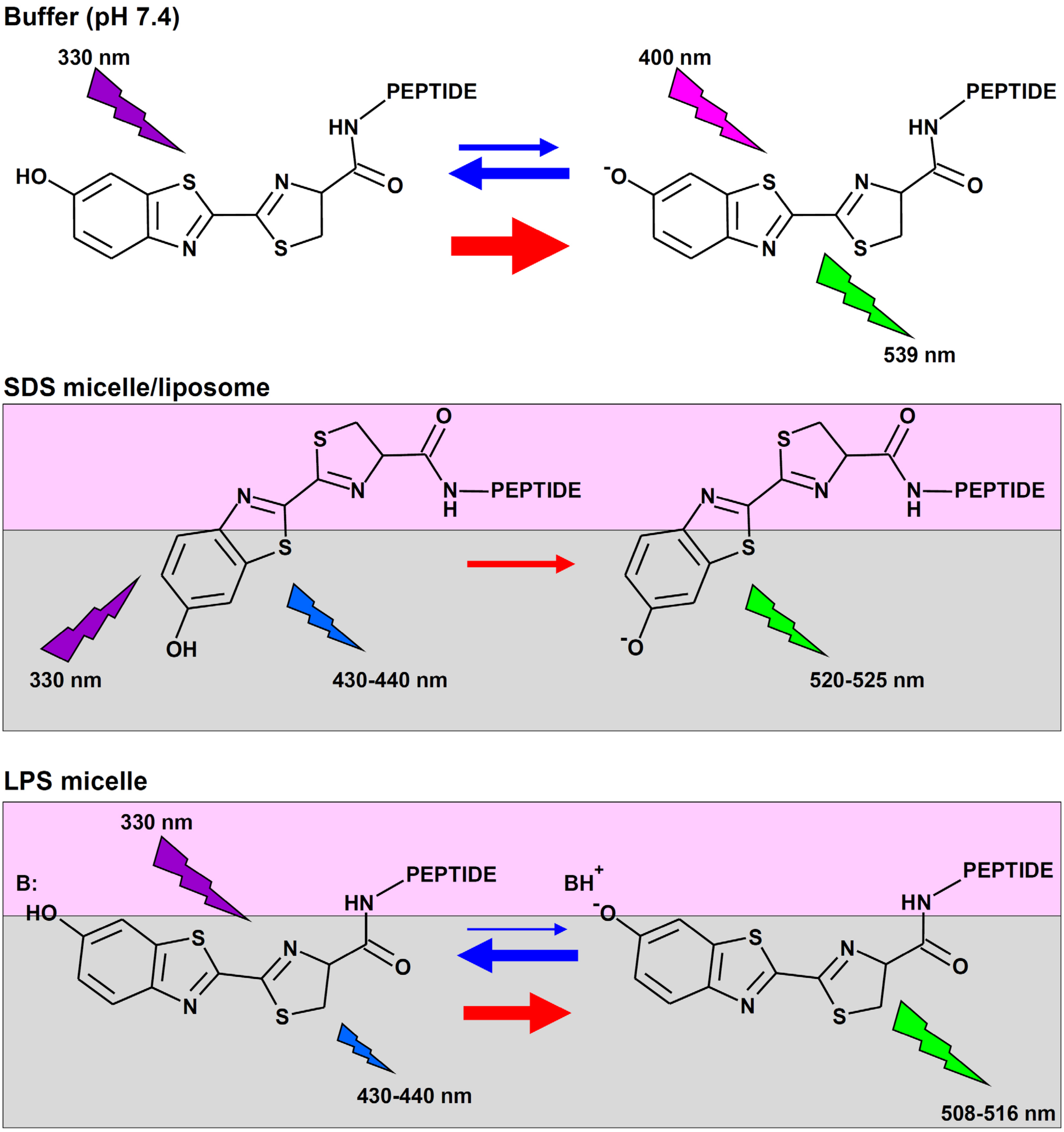

Figure S8. Possible orientations of Luc-labeled peptides bound to SDS micelles/liposomes or LPS. Gray and pink boxes represent, respectively, the inner hydrophobic region and the hydrophilic surface of liposomes and micelles. Blue and red arrows indicate acid-base equilibria and photoinduced ionization events, respectively. “B:” is a generic proton acceptor (either a solvent molecule or a group in the polar portion of the lipide A); the proton acceptor might be different in the case of the acid base equilibrium and the photoinduced ionization. In SDS micelles/liposomes the luciferin moiety is less buried than in LPS micelles but the hydroxyl group is better shielded from proton acceptors thus reducing the efficiency of the photoinduced dissociation and increasing the intensity of the blue fluorescence (430-440 nm).

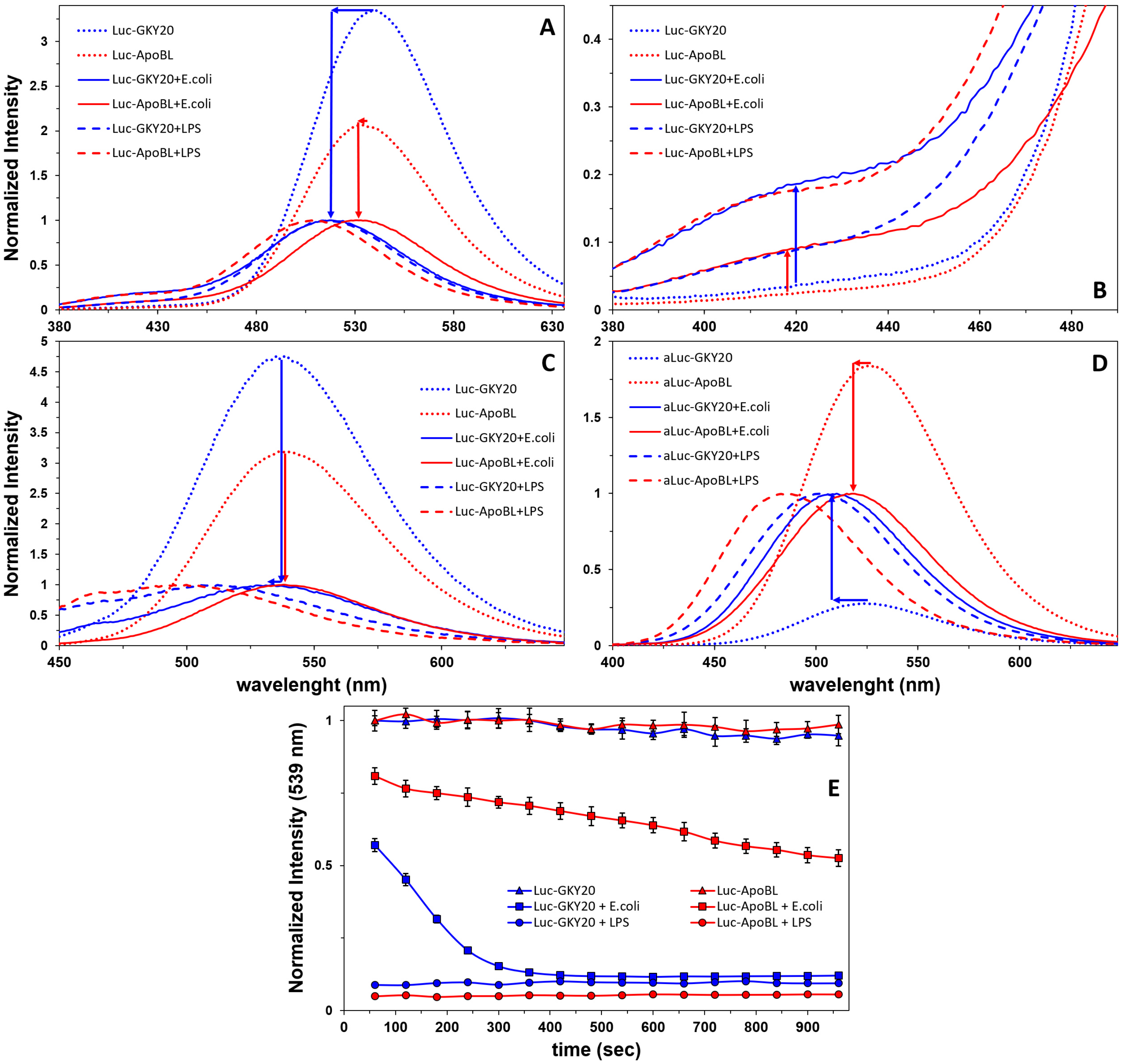

Figure S9. Emission spectra of labeled peptides in the presence of *E. coli* cells (0.1 O.D.). (**A**) Emission spectra of Luc-labeled peptides excited at 330 nm. (**B**) Close up of the blue region of the spectra in (A). (**C**) Emission spectra of Luc-labeled peptides excited at 400 nm. (**D**) Emission spectra of aLuc-labeled peptides excited at 363 nm. Spectra recorded in NaP (dotted lines) were normalized to the corresponding spectra recorded in the presence of *E. coli* cells. Arrows highlight the main changes with respect to NaP. (**E**) Variation of fluorescence (ex. 400 nm; em. 539 nm) as function of the incubation time.

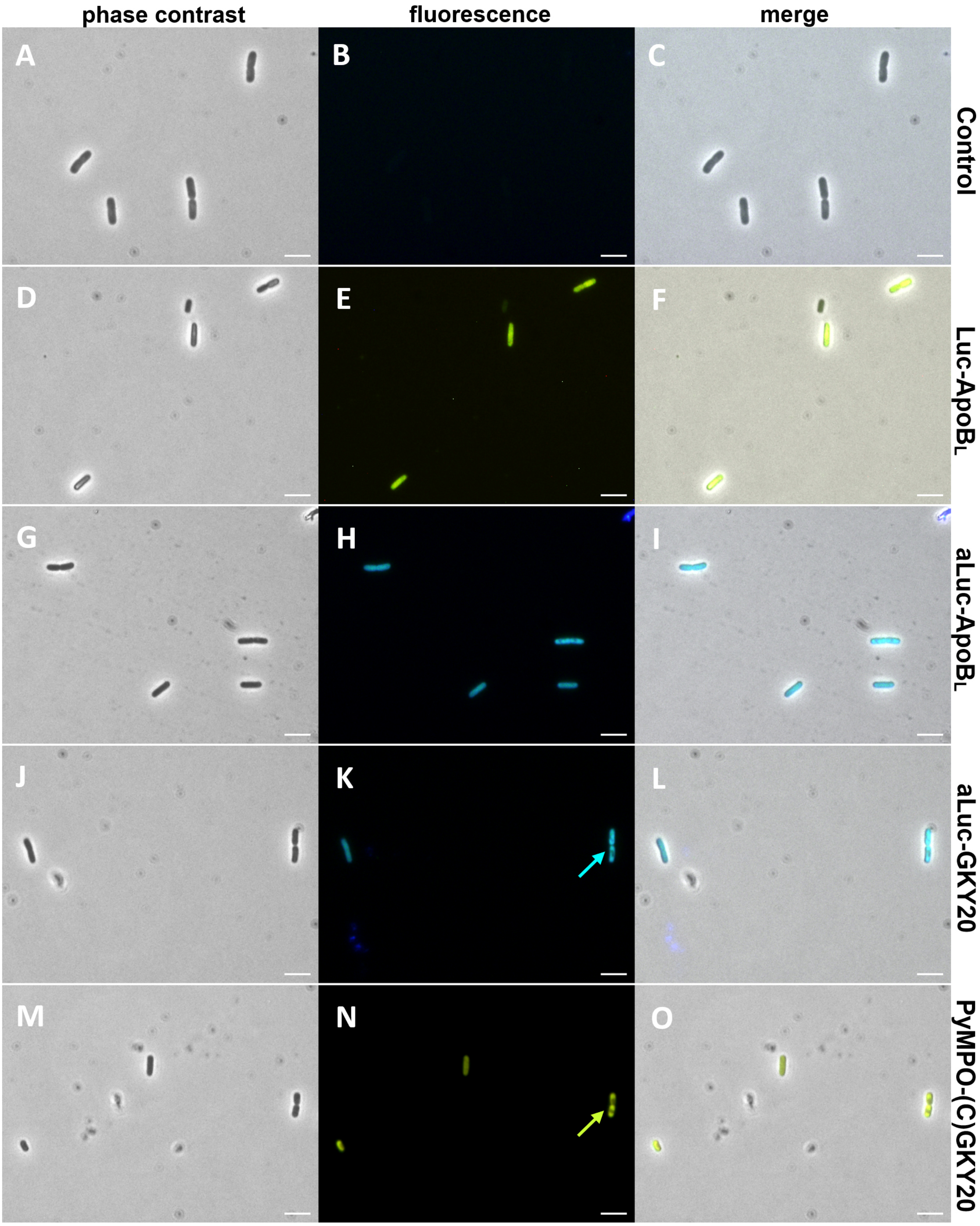

Figure S10. Fluorescence microscopy images of *E. coli* cells treated with labeled GKY20 and ApoBL. *E. coli* was incubated with peptides (3 μM) for 15 min and observed without further treatments. Cyan and yellow arrows highlight dividing cells with a heterogeneous staining. Bar, 2 μm.

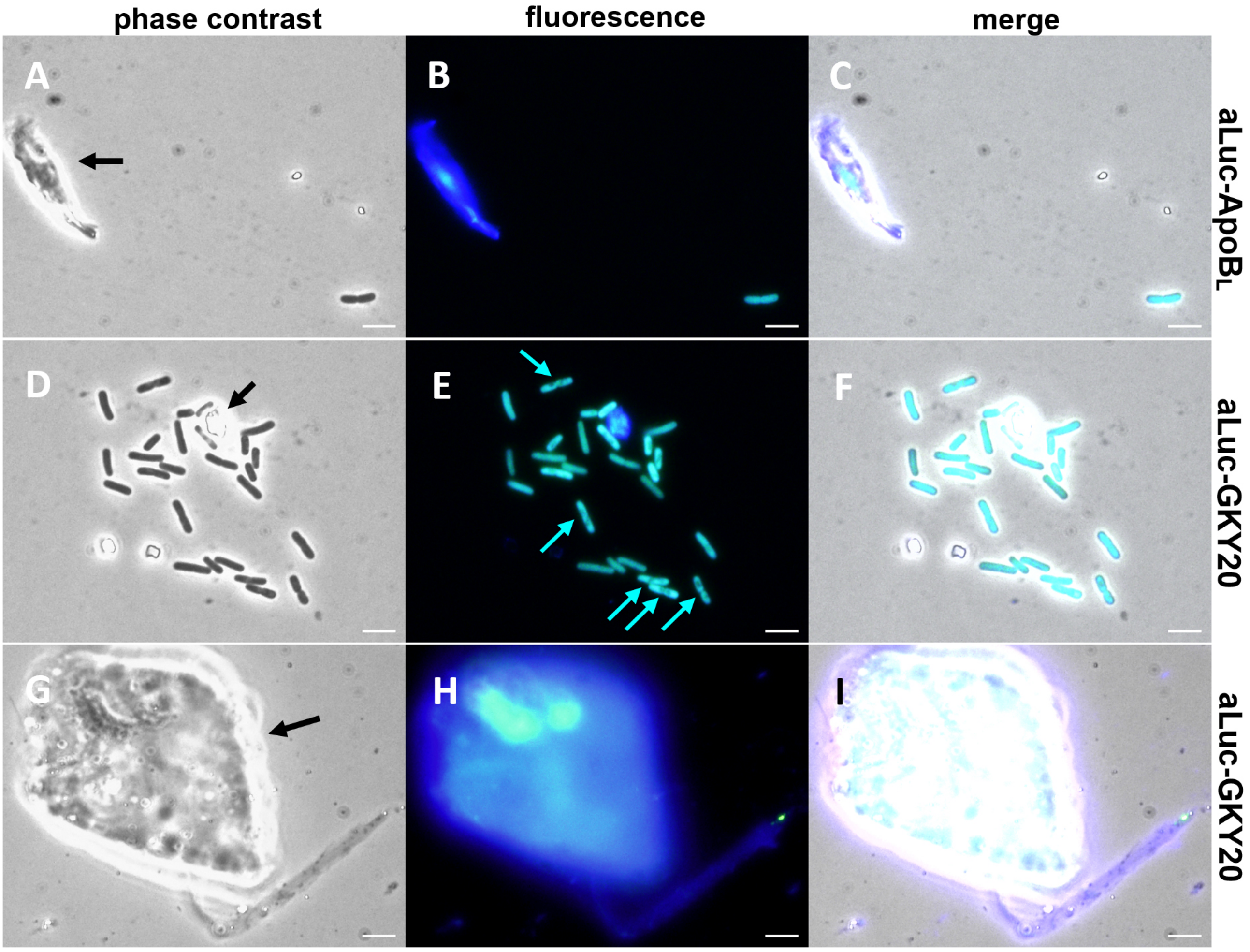

Figure S11. Fluorescence microscopy images of *E. coli* cells treated with labeled GKY20 and ApoBL. *E. coli* was incubated with peptides (3 μM) for 30 min (**A**–**F**) or 50 min (**G**–**I**). Cyan arrows highlight dividing cells with a heterogeneous staining. Black arrows highlight fluorescent aggregates. Bar, 2 μm.

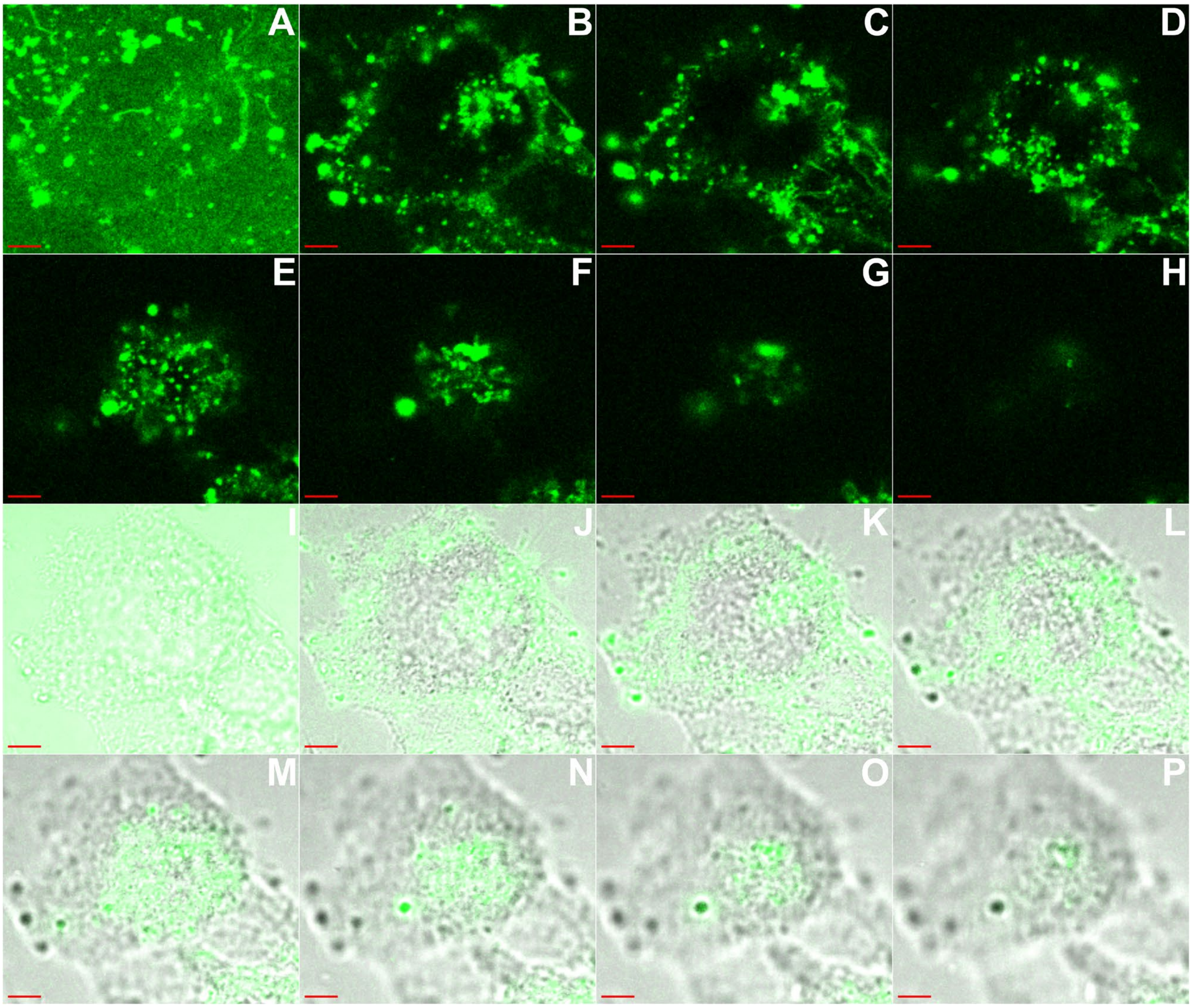

Figure S12. Z-stack CLSM images of HaCaT cells treated with aLuc-p53pAnt for 120 min. (**A–H**) Fluorescence of aLuc-p53pAnt. (**I–M**) Overlay between the bright field and the green channel. Panels are consecutive images taken at 2 μm increments. Images in (A) and (I) are the closest to bottom of the well. Scale bar = 5 μm. All the panels show a single cell representative of the culture. Most of the peptide is associated either to the bottom of the well (panel A and I), likely adsorbed to the glass directly or through binding to serum proteins, or to spots visible near the surface of the cell.

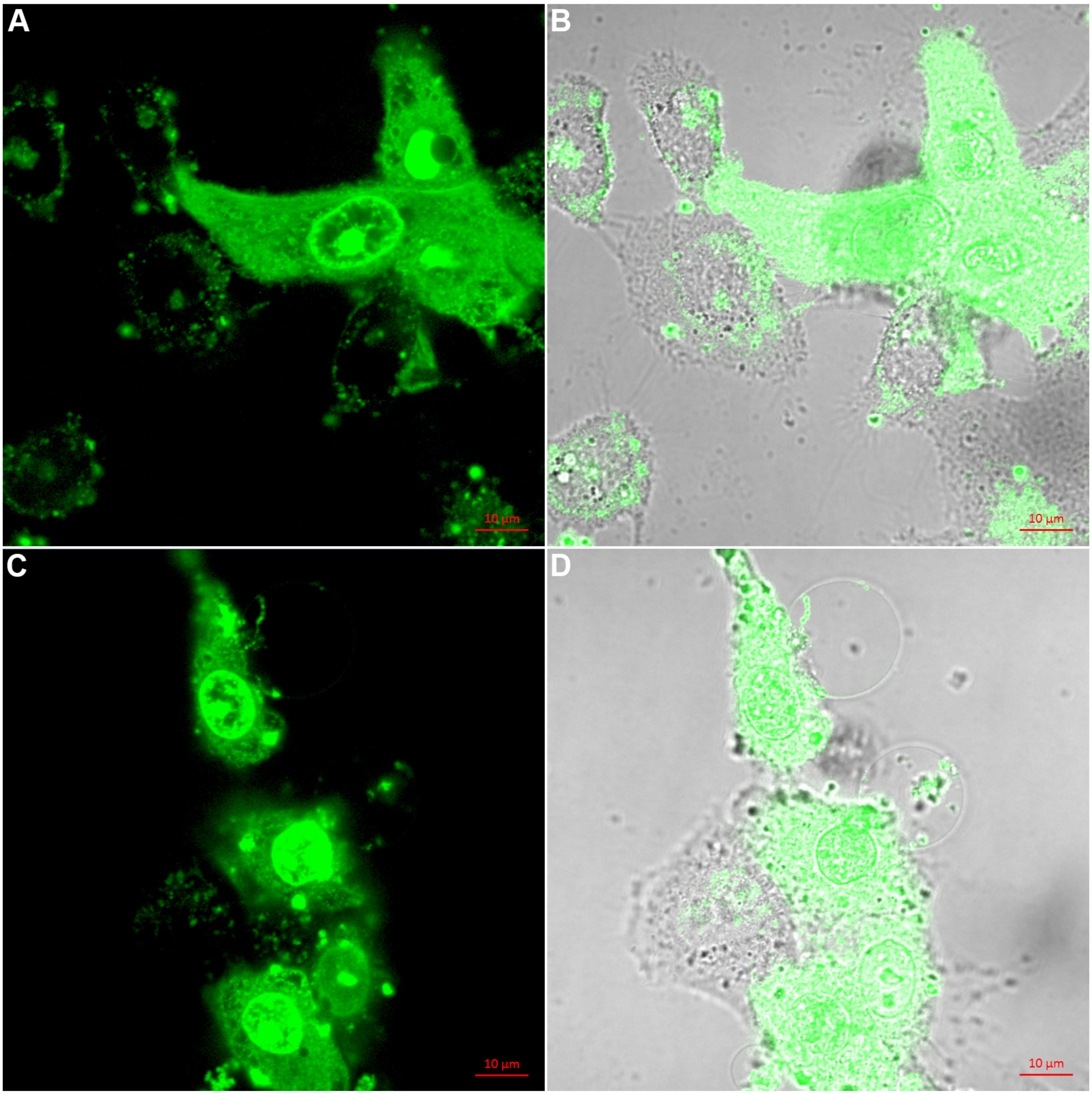

Figure S13. CLSM images of HaCaT cells treated with aLuc-p53pAnt for 120 min (**A**, **B**) and of Hela cells treated with aLuc-p53pAnt for 70 min (**C**, **D**). (B) and (D) show the overlay between the bright field and the green channel images shown in (A) and (C), respectively. Scale bar = 10 µm.

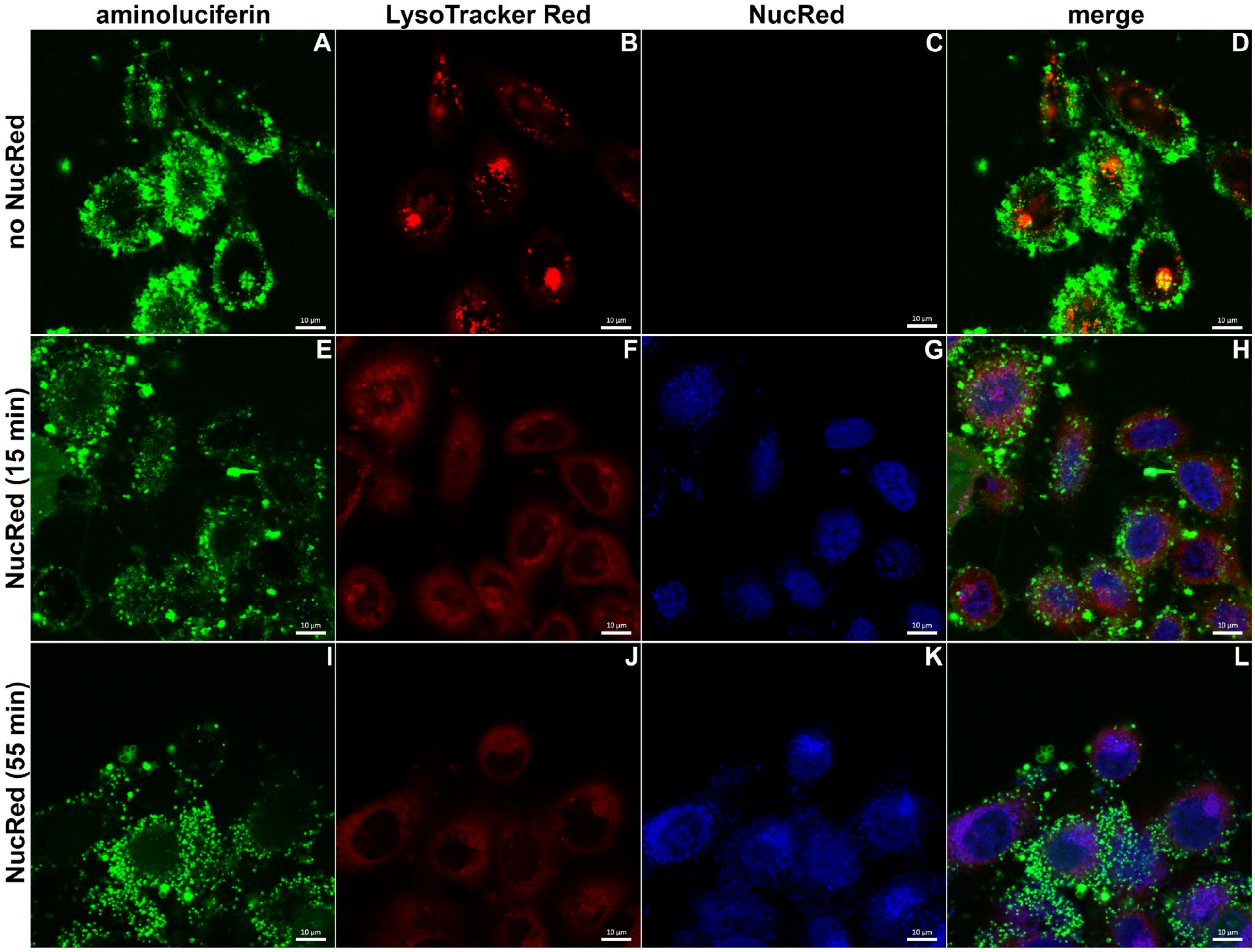

Figure S14. CLSM images of HaCaT cells treated with aLuc-p53pAnt. (**A**–**D**) Cells were incubated with the peptide for 75 min and with LysoTracker™ Red DND-99 for 15 additional minutes. (**E**–**H**) Cells were incubated with the peptide for 90 min and with LysoTracker™ Red DND-99 and NucRed™ Live 647 for 15 additional minutes. (**I**–**L**) Cells were co-incubated for 55 min with aLuc-p53pAnt, LysoTracker™ Red DND-99 and NucRed™ Live 647. Scale bar = 10 µm.

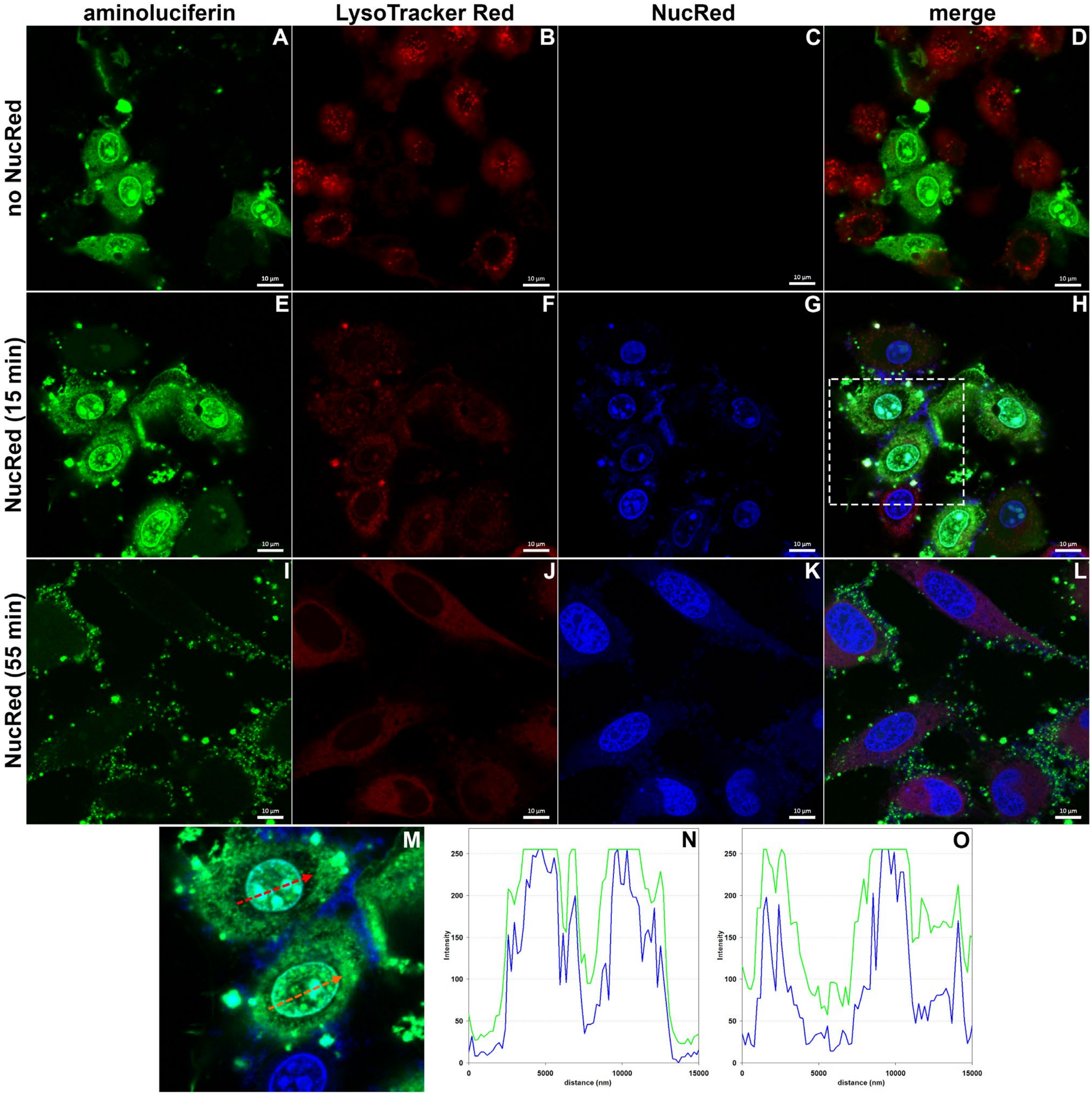

Figure S15. CLSM images of HeLa cells treated with aLuc-p53pAnt. (**A**–**D**) Cells were incubated with the peptide for 110 min and with LysoTracker™ Red DND-99 for 15 additional minutes. (**E**–**H**) Cells were incubated with the peptide for 105 min and with LysoTracker™ Red DND-99 and NucRed™ Live 647 for 15 additional minutes. (**I**–**L**) Cells were co-incubated for 55 min with aLuc-p53pAnt, LysoTracker™ Red DND-99 and NucRed™ Live 647. Scale bar = 10 µm. (**M**) Close up of the region highlighted by the dashed box in panel H. Both arrows correspond to 15 µm. (**N**, **O**) Analysis of the colocalization between the peptide (green curve) and NucRed (blue curve) across the red and orange arrows, respectively, in panel M.

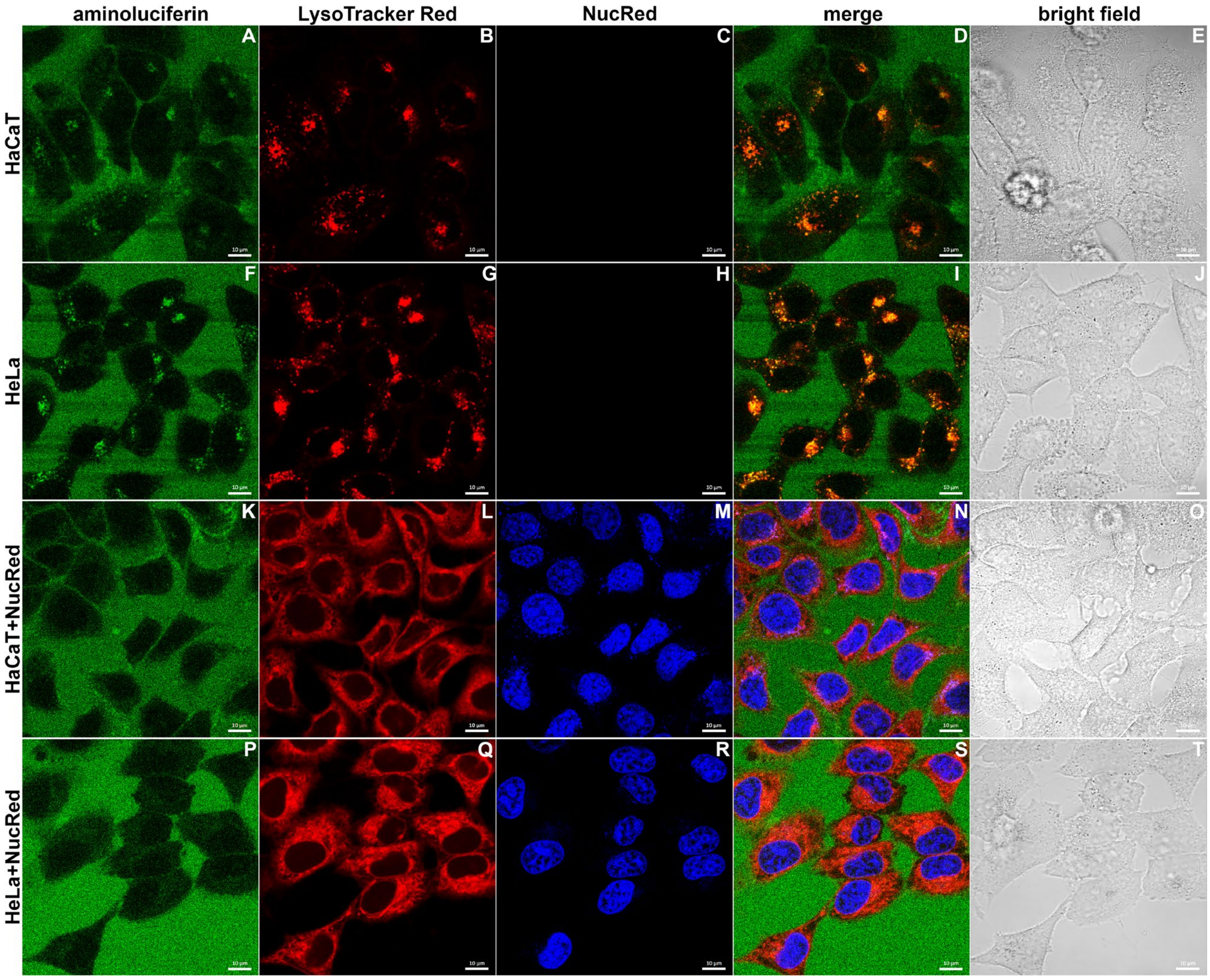

Figure S16. CLSM images of HaCaT and HeLa cells treated with aLuc-RGD. (**A**–**J**) Cells were incubated with the peptide for 90 min and with LysoTracker™ Red DND-99 for 15 additional minutes. (**K**–**T**) Cells were incubated with the peptide for 100 min and with LysoTracker™ Red DND-99 and NucRed™ Live 647 for 15 additional minutes. Scale bar = 10 μm.
